# Cancer and COVID-19 Susceptibility and Severity: A Two-Sample Mendelian Randomization and Bioinformatic Analysis

**DOI:** 10.3389/fcell.2021.759257

**Published:** 2022-01-24

**Authors:** Yiyin Zhang, Qijiang Mao, Yirun Li, Jiaxi Cheng, Qiming Xia, Guoqiao Chen, Peng Chen, Shengxi Jin, Duguang Li, Cheng Zhong, Jing Yang, Xiaoxiao Fan, Yuelong Liang, Hui Lin

**Affiliations:** ^1^ Department of General Surgery, Sir Run Shaw Hospital, School of Medicine, Zhejiang University, Hangzhou, China; ^2^ State Key Laboratory of Modern Optical Instrumentations, Centre for Optical and Electromagnetic Research, College of Optical Science and Engineering, International Research Center for Advanced Photonics, Zhejiang University, Hangzhou, China; ^3^ Zhejiang Engineering Research Center of Cognitive Healthcare, Sir Run Shaw Hospital, School of Medicine, Zhejiang University, Hangzhou, China

**Keywords:** COVID-19, cancer, Mendelian randomization, GWAS, ERAP2, lung adenocarcinoma

## Abstract

The clinical management of patients with COVID-19 and cancer is a Gordian knot that has been discussed widely but has not reached a consensus. We introduced two-sample Mendelian randomization to investigate the causal association between a genetic predisposition to cancers and COVID-19 susceptibility and severity. Moreover, we also explored the mutation landscape, expression pattern, and prognostic implications of genes involved with COVID-19 in distinct cancers. Among all of the cancer types we analyzed, only the genetic predisposition to lung adenocarcinoma was causally associated with increased COVID-19 severity (OR = 2.93, β = 1.074, se = 0.411, *p* = 0.009) with no obvious heterogeneity (Q = 17.29, *p* = 0.24) or symmetry of the funnel plot. In addition, the results of the pleiotropy test demonstrated that instrument SNPs were less likely to affect COVID-19 severity *via* approaches other than lung adenocarcinoma cancer susceptibility (*p* = 0.96). Leave-one-out analysis showed no outliers in instrument SNPs, whose elimination rendered alterations in statistical significance, which further supported the reliability of the MR results. Broad mutation and differential expression of these genes were also found in cancers, which may provide valuable information for developing new treatment modalities for patients with both cancer and COVID-19. For example, *ERAP2*, a risk factor for COVID-19-associated death, is upregulated in lung squamous cancer and negatively associated with patient prognosis. Hence, ERAP2-targeted treatment may simultaneously reduce COVID-19 disease severity and restrain cancer progression. Our results highlighted the importance of strengthening medical surveillance for COVID-19 deterioration in patients with lung adenocarcinoma by showing their causal genetic association. For these patients, a delay in anticancer treatment, such as chemotherapy and surgery, should be considered.

## Introduction

Coronavirus disease 2019 (COVID-19), which arises from severe acute respiratory syndrome coronavirus 2 (SARS-CoV-2) infection, can result in severe illnesses such as acute respiratory distress syndrome, multiorgan dysfunction syndrome, and consequent death, and it has become a public health emergency of international concern (Huang et al., 2020).

During the COVID-19 pandemic, the clinical management of patients with cancer is a Gordian knot that has been discussed broadly but has not reached a consensus (Moujaess et al., 2020). Two observational studies from China revealed that patients with cancer were more susceptible to COVID-19 and its relevant severe complications (Dai et al., 2020; Liang et al., 2020). Several potential explanations may account for the vulnerability to COVID-19 among patients with cancer. First, most chemotherapy induces myelosuppression and an immunosuppressive condition in patients, which increases the risk of succumbing to COVID-19 and the occurrence of adverse events. Second, the physiological function of patients with cancer is dramatically undermined, either attributed to disease progression or posttreatment side effects, which may synergistically lead to an unfavorable prognosis from COVID-19 in patients with cancer (Diao et al., 2020). Third, immune checkpoint inhibitors (ICIs) have been widely used in the treatment of multiple cancers. ICIs could induce immune-related pneumonitis followed by lung injury, which could enhance the risk of developing severe COVID-19 pneumonia. However, some studies also reported that CoV-2 infection causes functional exhaustion of CTLs and NK cells with significantly higher levels of exhaustion markers such as programmed death-1 (PD-1) than healthy controls (Zheng et al., 2020), suggesting that the use of ICIs may activate the anti-COVID-19 ability in the host and improve the patient prognosis. Two ongoing clinical trials will demonstrate the pros and cons of adopting ICIs in COVID-19 treatment (NCT04343144 and NCT04333914).

These different findings revealed the close relationship between cancer and COVID-19 susceptibility and severity, and it is difficult to speculate on their causal relationship given the unavoidable bias derived from unmeasured confounding factors in traditional observational studies. Hence, we introduced Mendelian randomization (MR) to investigate the causal association between cancers and COVID-19 susceptibility and severity.

MR is an increasingly acknowledged statistical method that uses genetic variants to determine whether an observational association between a risk factor and an outcome is consistent with a causal effect (Emdin et al., 2017). Individuals who carry the variant and those who do not are followed up until the development of an outcome of interest. Because these genetic variants are typically unrelated to confounding factors, differences in the outcome between those who carry the variant and those who do not could be attributed to the difference in the risk factor, which makes verification of the causal association accessible (Sekula et al., 2016). MR is based on 3 assumptions: (1) the genetic variant is associated with the risk factor; (2) the genetic variant is not associated with confounders; and (3) the genetic variant influences the outcome only through the risk factor. Given the difficulty of concurrently measuring the exposure and outcome traits in the same cohort, as an alternative, summary-level data from different genome-wide association study (GWAS) consortia can be used to carry out MR analyses, taking gene exposure measures from one GWAS and gene outcome measures from another GWAS (two-sample MR) (Lawlor, 2016).

The present study investigated the causal association between cancer and COVID-19 susceptibility and severity using two-sample MR analysis. Moreover, we also explored the mutation landscape, expression pattern, and prognostic implications of genes involved with COVID-19 in distinct cancers.

## Materials and Methods

### Summarize Observational Studies About COVID-19 and Cancers and Calculate the E-Value

A systematic retrieval of studies pertaining to COVID-19 and cancers was conducted using the following terms: (COVID-19[Title/Abstract] OR COVID-19[Title/Abstract] OR SARS-CoV-2[Title/Abstract]) AND (cancer [Title/Abstract] OR tumor[Title/Abstract] OR malignancy[Title/Abstract]).

The E-value is defined as the minimum strength of an association that an unmeasured confounder would need to have with both the exposure and the outcome to fully explain away a specific exposure–outcome association, conditional on the measured covariates (Blum et al., 2020; VanderWeele and Ding, 2017). A large E-value implies that considerable unmeasured confounding would be needed to explain away an effect estimate, while a small E-value implies little unmeasured confounding would be needed to explain away an effect estimate. The E-value was calculated on a website (https://www.evalue-calculator.com/), and it could be calculated for an observed risk ratio (denoted RR) by E-value = RR + √[RR*(RR − 1)]. If the original risk ratio is below 1, then one first takes the inverse before applying the E-value formula (Mathur et al., 2018). Hence, we calculated the E-value to estimate the bias from unmeasured confounders in the existing observational studies focused on the relationship between cancer and COVID-19 sensitivity and severity.

### Identify Cancer-Associated Genetic Variants

Initially, we selected 10 cancers with no obvious genetic proposition by sex to maximally reduce the bias derived from sex differences (glioma, squamous lung cancer, lung adenocarcinoma, melanoma, lymphoid leukemia, hepatocellular carcinoma, colorectal carcinoma, kidney cancer, gastric cancer, and pancreatic cancer). Then, we identified the SNPs that were significantly associated with each cancer with the threshold value *p* < 5e-8 in the GWAS Catalog repository (https://www.ebi.ac.uk/gwas/) (MacArthur et al., 2017). SNPs without the required information for computing the MR analysis, such as β(se), effect alleles, and their frequency, were eliminated. In addition, we only included SNPs associated with cancer susceptibility, and other traits, such as the patients’ overall survival time or chemotherapy resistance, were not within our scope.

### Select GWAS Involved With COVID-19 Susceptibility and Severity

The meta-analysis results of SNP-based association analysis pertaining to COVID-19 susceptibility and severity were obtained from the COVID-19 Host Genetics Initiative (https://www.covid19hg.org/) (Release 4) (COVID-19 Host Genetics Initiative, 2020). The phenotype “COVID vs. laboratory/self-reported negative” assessed the effect of SNPs on COVID-19 susceptibility, while the phenotype “very severe respiratory confirmed COVID versus not hospitalized COVID” was selected to evaluate the effect of SNPs on COVID-19 severity.

### Procedures for Two-Sample MR Analysis

For standard two-sample MR, it is important to ensure that the instruments for the exposure are independent. Among those SNPs that have linkage disequilibrium (LD) R-squares above the specified threshold, only the SNP with the lowest *p*-value will be retained. We pruned all SNPs in LD using the default cutoff value recommended by the “MR-base” platform. The clumping distance (kb) was set as 10,000 kb and *R* ^2^ was 0.01. By default, if a particular requested SNP is not found in the outcome GWAS, then a SNP (proxy) in the LD with the requested SNP (target) will be searched for instead. Once the exposure and outcome data are obtained, the next step is to harmonize the effects of the instrumental variants, which refers to the effect of a SNP on the exposure, and the effect of that SNP on the outcome must correspond to the same allele. For inferable palindromic SNPs, we tried to infer the forward strand alleles using allele frequency information. Noninferable palindromic SNPs referring to the allele frequency no longer provide information about the strand. Such SNPs would be discarded. This is done for any palindromic SNPs that have minor allele frequencies above 0.42.

Once the exposure and outcome data were harmonized, the effects and standard errors for each instrument SNP were available for the exposure and outcome traits. Such information could be utilized to perform MR analysis. Inverse-variance weighted (IVW) estimation is a classic method to pool the MR effects of each instrument SNP.

The variance term was calculated as $\frac{se{\left({\hat{\beta }}_{Yj}\right)}^{2}}{{\hat{\beta }}^{2}}$ , and the pooled fixed-effect inverse-variance weighted estimate ( ${\hat{\theta }}_{IVW}$ ) was calculated as ${\hat{\theta }}_{IVW}=\frac{\sum_{j}{\hat{\beta }}_{Yj}{\hat{\beta }}_{Xj}se{\left({\hat{\beta }}_{Yj}\right)}^{-2}}{\sum_{j}{\hat{\beta }}^{2}se{\left({\hat{\beta }}_{Yj}\right)}^{-2}}$ . In addition, other methods for two-sample MR, including MR Egger, weighted mode, weighted median, and simple mode, were also adopted for reference. Each method differs in theory and has distinct strengths. The weighted mode introduces an extra element similar to IVW and the weighted median, weighting each SNP’s contribution to the clustering by the inverse variance of its outcome effect (Hartwig et al., 2017). A median-based estimator is an alternative approach that takes the median effect of all available SNPs, which has the advantage that only half of the SNPs need to be valid instruments for unbiased causal effect estimates. The weighted median estimate allows for stronger SNPs to contribute more toward the estimate and they can be acquired by weighting the contribution of each SNP by the inverse variance of its association with the outcome (Bowden et al., 2016a). MR–Egger adapts the IVW analysis by allowing a nonzero intercept, allowing the net-horizontal pleiotropic effect across all SNPs to be unbalanced or directional (Bowden et al., 2016b). Hence, these methods should be considered, especially when the basic assumptions behind MR, such as the absence of a horizontal pleiotropic effect and heterogeneity, are not well satisfied.

Heterogeneity in the causal effects among instruments is a marker of the potential violations of instrumental variants’ assumptions. Heterogeneity could be assessed for the IVW and Egger estimates, and this can be used to navigate between models of horizontal pleiotropy (Bowden et al., 2017). In addition, we depicted funnel plots to visualize any heterogeneity of effect estimates, where the causal effect estimates for each SNP were depicted on the *x*-axis and the inverse standard error (a measure of instrumental strength) for the association was represented on the *y*-axis. Asymmetry about the vertical line is indicative of the heterogeneity.

To evaluate the robustness of the MR effect estimates and identify any potential outliers, each instrument SNP was sequentially eliminated from the analysis (referred to as a leave-one-out analysis). If the precision and direction of the association between the cancer-predicting SNPs and COVID-19 susceptibility and severity remained largely unaltered, then the results were probably not driven by any outliers. All analyses were performed using the R package “TwoSampleMR” (version 0.5.5). Bonferroni correction was performed by dividing the *p*-value of 0.05 by the number of testing methods (here, the threshold should be 0.05/5 = 0.01).

### Investigate the Expression Pattern and Prognostic Implications of Genes Involved in COVID-19 Susceptibility and the Severity in Cancers

The omics and clinical data of cancers derived from the primary organs that were the same as the cancer types investigated in the MR analysis were collected from The Cancer Genome Atlas (TCGA) (https://www.cancer.gov/). The whole exome sequencing (WES) and copy number variation (CNV) data of each cancer were integrated and processed through cBioPortal (www.cbioportal.org) (Cerami et al., 2012; Gao et al., 2013). Given the lack of adjacent normal samples in the TCGA database, we incorporated the transcriptome data of the corresponding normal organs from the Genotype-Tissue Expression (GTEx) datasets. Using the combined transcriptome data from TCGA and GTEx, we compared the differential expression of genes associated with COVID-19 between tumor tissues and normal tissues using the cutoff of logFC >1 and adjusted *p*-value <0.01. The association between the gene expression level and the patient prognosis was also investigated. The patients were divided into two groups based on the median gene expression level, and OS and DFS were compared between the two groups using Kaplan–Meier methods. The log-rank test was performed to evaluate the statistical significance of the survival differences (the cutoff was set as *p* < 0.05). The core code for performing the MR analysis is attached in the Supplementary Method.

## Results

### A Summary of the Observational Studies Pertaining to the Cancer and COVID-19 Susceptibility and Severity

Many studies have investigated the clinical characteristics of COVID-19 patients with cancer (Table 1). However, most of them failed to compare the susceptibility and severity of COVID-19 between patients with or without cancer. Only three studies provided direct evidence to show that patients with cancer appear more vulnerable to SARS-CoV-2. Although a multivariable model was applied to adjust common confounding factors when evaluating the risk for severe COVID-19 manifestations, many potential unmeasured confounders may still exist and affect the reliability of the results (Dai et al., 2020; Liang et al., 2020; Mehta et al., 2020). Hence, we calculated the E-value to estimate the possibility that unmeasured confounders interrupted the results. The E-values for the observed association between cancer and COVID-19-induced mortality, rates of ICU admission, severe or critical symptoms, and deterioration were 4.11, 2.76, 2.73, and 4.18, respectively, which were not large enough to eliminate the possibility of bias on the causality derived from unmeasured confounders.

**TABLE 1:** A summary of observational studies pertaining to COVID-19 and cancers.

Study	Country	Patients number	Main conclusion	PMID	E_value
Dai et al.	China	105 COVID-19 patients with cancer versus 536 age-matched noncancer patients confirmed with COVID-19	Patients with cancer appear more vulnerable to SARS-CoV-2 outbreak	32345594	Motality:4.11; rates of ICU admission:2.76; severe or critical symptom:2.73
Liang et al.	China	1,590 COVID-19 patients	Patients with cancer were more likely to succumb to COVID-19 compared with the general population (1% vs. 0.29%).	32066541	COVID-19 deterioration:4.18
Garassino et al.	Italy	200 COVID-19 patients with thoracic malignancy	High mortality and low admission to intensive care in patients with thoracic cancer.	32539942	\
Kuderer et al.	America, Canada, and Spain	928 COVID-19 patients with cancer	Cancer type is not associated with the 30-day all-cause mortality of COVID-19.	32473681	\
Onder et al.	Italy	355 patients died from COVID-19	Patients who died from COVID-19 in Italy found that 20.3% of the deceased had active cancer	32203977	\
Signorelli et al.	Italy	\	The pooled prevalence of the virus in patients with cancer is as high as 2%–3%, suggesting that cancer patients are largely over-represented among fatalities.	32275287	\
Metha et al.	America	218 COVID-19 patients with malignancy	A total of 61 (28%) patients with cancer died from COVID-19 with a case fatality rate (CFR) of 37% (20/54) for hematologic malignancies and 25% (41/164) for solid malignancies. Six of 11 (55%) patients with lung cancer died from COVID-19 disease.	32357994	\
Robilotti et al.	America	423 COVID-19 patients with cancer	Roughly 40% (169/423) of the patients with cancer diagnosed with COVID-19 were admitted, 20% (85/423) developed severe respiratory illness, and 9% (38/423) died	32581323	\

### Identification of Instrumental Variables for Cancer and the Effects of These Instrument SNPs on COVID-19 Susceptibility and Severity

**FIGURE 1:**
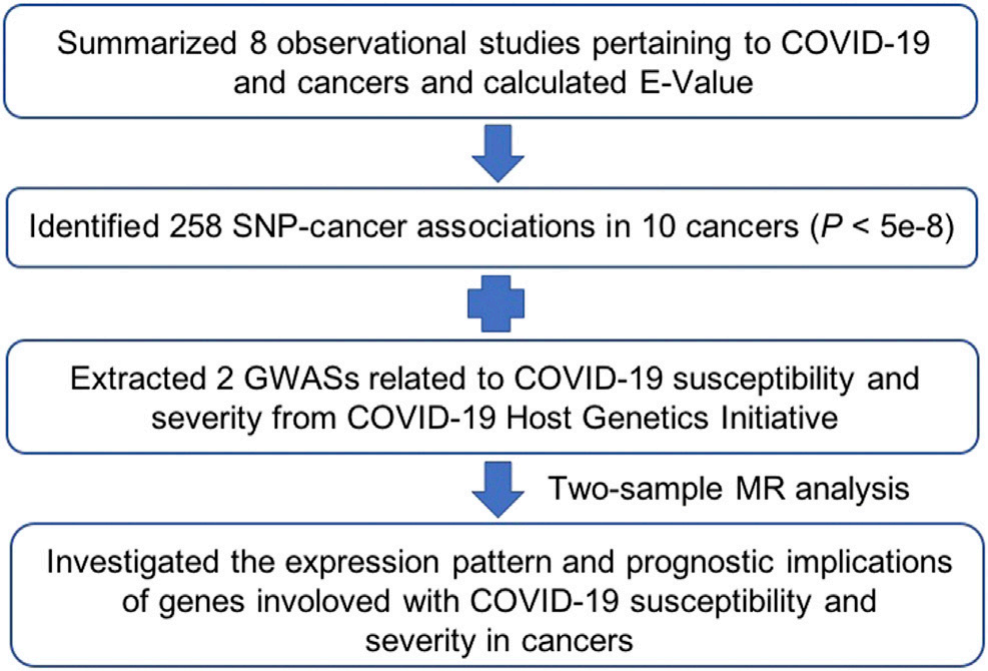
Schematic diagram illustrating the Mendelian randomization (MR) procedure used in the main analysis.

A total of 258 GWASs associated with the effect of SNPs on 10 cancers were included for screening instrumental variables (Figure 1; Supplementary Tables S1–S2). A total of 9,536,812 participants across distinct regions were included in these studies. We obtained access to 2 GWASs involved in COVID-19 susceptibility and severity. The GWAS data of 24,057 participants who were diagnosed with COVID-19 and 218,062 normal participants with laboratory or self-reported negative results of COVID-19 testing were enrolled in the present study, which was contributed to by 23 independent studies. In addition, the GWAS data of 4 cohorts, including 269 COVID-19 patients with severe respiratory symptoms and 688 nonhospitalized COVID-19 participants, were analyzed to identify SNPs strongly associated with COVID-19 severity (Supplementary Table S3). When these data were collected, we further obtained the effects of cancer-associated SNPs on COVID-19 susceptibility and severity and performed MR analysis. The SNPs used in the MR analysis and their effects are listed in Supplementary Table S4.

### Study of the Causal Association Between the Genetic Predisposition to Cancer and COVID-19 Susceptibility

**FIGURE 2:**
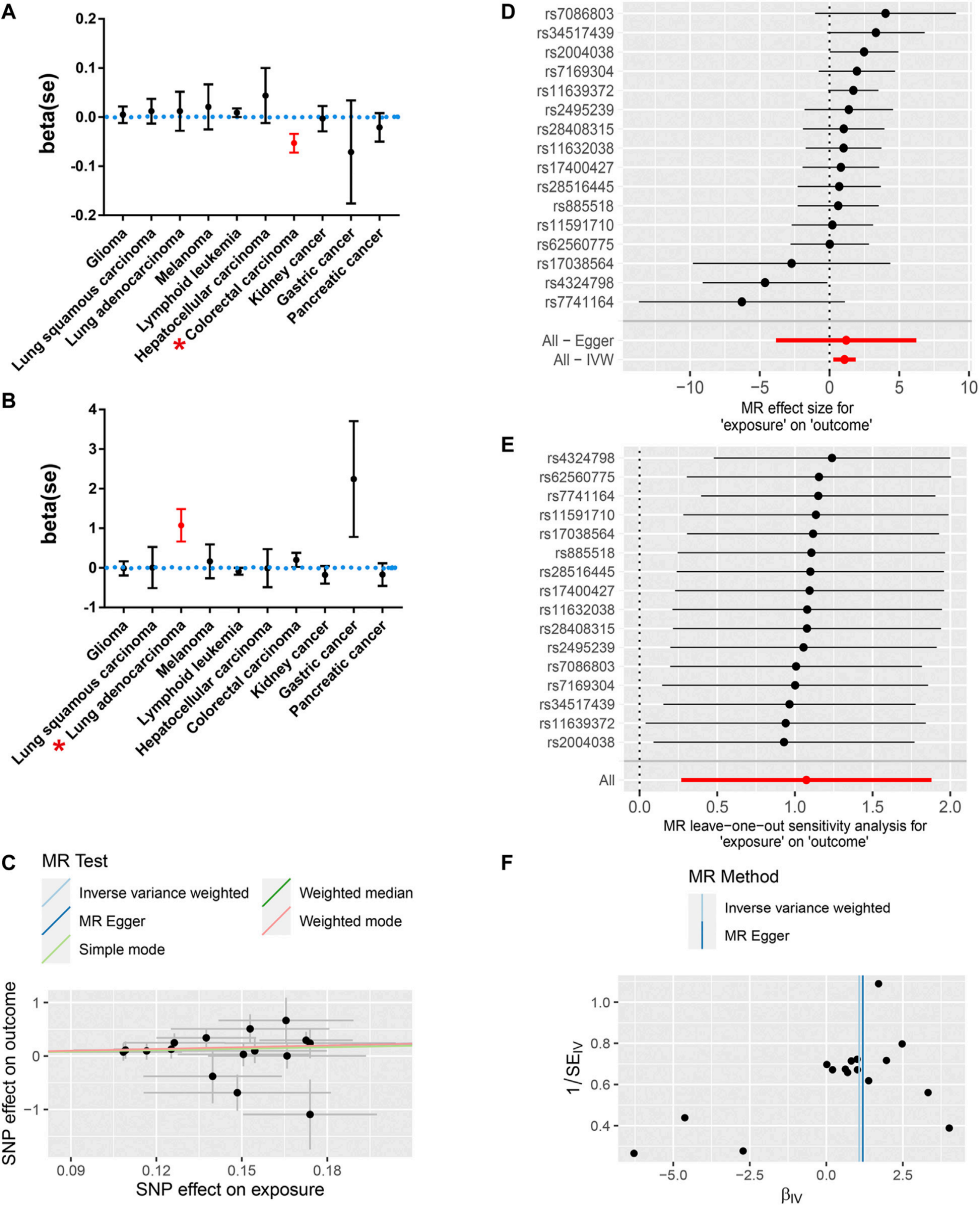
The MR results revealed a causal association between a genetic predisposition to cancers and COVID-19 susceptibility and severity. **(A)** A genetic predisposition to colorectal cancer is causally associated with decreased susceptibility to COVID-19. **(B)** The genetic predisposition to lung adenocarcinoma is causally related to increased COVID-19 severity. Red* indicates statistical significance (*p* < 0.05). **(C)** The scatter plot shows a positive correlation between the genetic predisposition to lung adenocarcinoma and an increased risk for severe COVID-19. **(D)** The MR effect for each SNP and their pooled effects. **(E)** Leave-one-out analysis showed no outliers, potentially leading to an alteration of the MR results. **(F)** The symmetry of the funnel plot showed no obvious pleiotropy in either the IVW or Egger MR model.

Among all of the cancer types we analyzed, only a genetic predisposition to colorectal cancer was causally associated with COVID-19 susceptibility (Figure 2A; Table 2). Specifically, an increased genetic predisposition to colorectal cancer could decrease the risk of succumbing to COVID-19 (β = −0.053, se = 0.019, *p* = 0.005). No obvious heterogeneity was detected by Q statistics (Q = 91.01, *p* = 0.833; Supplementary Table S5) or the symmetry of the funnel plot. In addition, the results of the pleiotropy test demonstrated that instrument SNPs were less likely to affect COVID-19 susceptibility *via* approaches other than colorectal cancer susceptibility (*p* = 0.680; Supplementary Table S6). Leave-one-out analysis showed no outliers in instrument SNPs, whose elimination caused alterations in statistical significance, which further supported the reliability of the MR results (Supplementary Table S7).

**TABLE 2:** Two-sample Mendelian randomization analysis for COVID-19 susceptibility and severity across 10 distinct cancers.

MR method	Glioma susceptibility		Glioma severity			
	Beta	se	*p*-value	Beta	se	*p*-value
MR Egger	−0.010	0.032	0.761	−0.053	0.331	0.876
Weighted median	−0.008	0.024	0.723	−0.168	0.201	0.404
Inverse variance weighted	0.005	0.017	0.778	−0.012	0.178	0.947
Simple mode	0.004	0.043	0.930	−0.038	0.373	0.920
Weighted mode	−0.021	0.027	0.463	−0.234	0.212	0.291
Squamous cell lung carcinoma susceptibility				Squamous cell lung carcinoma severity		
MR Egger	−0.046	0.047	0.344	0.948	1.049	0.387
Weighted median	−0.005	0.034	0.886	0.153	0.522	0.769
Inverse variance weighted	0.012	0.025	0.620	0.008	0.519	0.988
Simple mode	0.093	0.057	0.126	−2.208	1.201	0.093
Weighted mode	−0.015	0.034	0.673	0.814	0.709	0.275
Lung adenocarcinoma susceptibility				Lung adenocarcinoma severity		
MR Egger	0.133	0.254	0.608	1.194	2.567	0.649
Weighted median	−0.032	0.054	0.551	1.016	0.505	0.044*
Inverse variance weighted	0.012	0.040	0.771	1.074	0.411	0.009*
Simple mode	−0.027	0.088	0.767	0.907	0.697	0.213
Weighted mode	−0.029	0.072	0.694	1.101	0.634	0.103
Melanoma susceptibility				Melanoma severity		
MR Egger	0.077	0.125	0.558	0.667	1.430	0.655
Weighted median	0.062	0.055	0.264	0.457	0.551	0.407
Inverse variance weighted	0.021	0.046	0.642	0.166	0.429	0.699
Simple mode	0.058	0.087	0.526	0.584	0.908	0.538
Weighted mode	0.072	0.074	0.361	0.781	0.864	0.392
Lymphoid leukemia susceptibility				Lymphoid leukemia severity		
MR Egger	0.010	0.021	0.638	−0.503	0.199	0.018*
Weighted median	0.011	0.013	0.394	−0.129	0.129	0.318
Inverse variance weighted	0.009	0.009	0.338	−0.086	0.086	0.318
Simple mode	0.013	0.019	0.493	−0.047	0.187	0.805
Weighted mode	0.008	0.014	0.565	−0.119	0.139	0.402
Hepatocellular carcinoma susceptibility				Hepatocellular carcinoma severity		
MR Egger	−0.060	0.203	0.816	1.384	1.447	0.514
Weighted median	0.041	0.032	0.208	0.096	0.280	0.730
Inverse variance weighted	0.044	0.056	0.428	−0.009	0.482	0.986
Simple mode	0.136	0.094	0.285	0.314	0.381	0.497
Weighted mode	−0.010	0.036	0.800	0.112	0.322	0.762
Colorectal cancer susceptibility				Colorectal cancer severity		
MR Egger	−0.068	0.042	0.104	0.919	0.405	0.025*
Weighted median	−0.047	0.032	0.144	0.355	0.284	0.211
Inverse variance weighted	−0.053	0.019	0.005*	0.203	0.179	0.256
Simple mode	−0.069	0.062	0.269	0.469	0.647	0.471
Weighted mode	−0.053	0.038	0.172	0.567	0.392	0.152
Kidney cancer susceptibility				Kidney cancer severity		
MR Egger	−0.022	0.045	0.643	−0.274	0.360	0.480
Weighted median	0.012	0.032	0.702	−0.255	0.287	0.373
Inverse variance weighted	−0.003	0.026	0.898	−0.177	0.222	0.424
Simple mode	0.000	0.044	0.993	−0.215	0.331	0.541
Weighted mode	0.002	0.036	0.956	−0.259	0.264	0.365
Gastric cancer susceptibility				Gastric cancer severity		
MR Egger	−0.607	0.862	0.609	\		
Weighted median	−0.012	0.099	0.903	\		
Inverse variance weighted	−0.071	0.105	0.499	2.244	1.464	0.125
Simple mode	0.041	0.138	0.795	\		
Weighted mode	0.050	0.157	0.781	\		
Pancreatic cancer susceptibility				Pancreatic cancer severity		
MR Egger	−0.150	0.091	0.120	−0.996	0.940	0.307
Weighted median	−0.013	0.038	0.726	0.392	0.383	0.305
Inverse variance weighted	−0.021	0.029	0.464	−0.170	0.287	0.555
Simple mode	0.011	0.072	0.886	0.492	0.648	0.460
Weighted mode	−0.004	0.074	0.954	0.536	0.547	0.342

### The Causal Association Between Genetic Susceptibility to Cancer and COVID-19 Severity

Then, we further analyzed the causal association between genetic predisposition to cancers and COVID-19 severity. Among all of the cancer types we analyzed, only genetic susceptibility to lung adenocarcinoma was causally associated with COVID-19 severity (Figure 2B; Table 2). Specifically, an increased genetic predisposition to lung adenocarcinoma could increase the risk of succumbing to severe COVID-19 (Figures 2C,D; OR = 2.93, β = 1.074, se = 0.411, *p* = 0.009). No obvious heterogeneity was detected by Q statistics (Q = 17.29, *p* = 0.24; Supplementary Table S5) or the symmetry of the funnel plot (Figure 2F). In addition, the results of the pleiotropy test demonstrated that instrument SNPs were less likely to affect COVID-19 severity *via* approaches other than lung adenocarcinoma cancer susceptibility (*p* = 0.96; Supplementary Table S6). Leave-one-out analysis showed no outliers in instrument SNPs, whose elimination resulted in alterations in statistical significance, which further supported the reliability of the MR results (Figure 2E; Supplementary Table S7). The causal relationship between genetic susceptibility to lung adenocarcinoma and COVID-19 severity was also confirmed in the “weighted median” model, which allows for stronger SNPs to contribute more toward the estimate (β = 1.016, se = 0.505, *p* = 0.044). To test whether the heterogeneity was derived from the differences among the cohorts, we performed subgroup analysis by only using the SNPs in the study by McKay et al. (2017), which is the largest lung adenocarcinoma GWAS cohort available in the GWAS catalog. As expected, the result of this subgroup analysis still demonstrated that the genetic predisposition to lung adenocarcinoma was causally related to an increased risk for severe COVID-19 (IVW model, β = 1.299, se = 0.410, *p* = 0.002) without any obvious heterogeneity or horizontal pleiotropy (Q = 0.42, *p* = 0.88; *p* = 0.79). To further support our conclusion, we performed MR analysis based on hospitalized and non-hospitalized COVID-19 cohort and found a similar conclusion (β = 0.28, se = 0.15, *p* = 0.05).

### Expression Pattern and Prognostic Implications of Genes Involved in COVID-19 in Cancers

**FIGURE 3:**
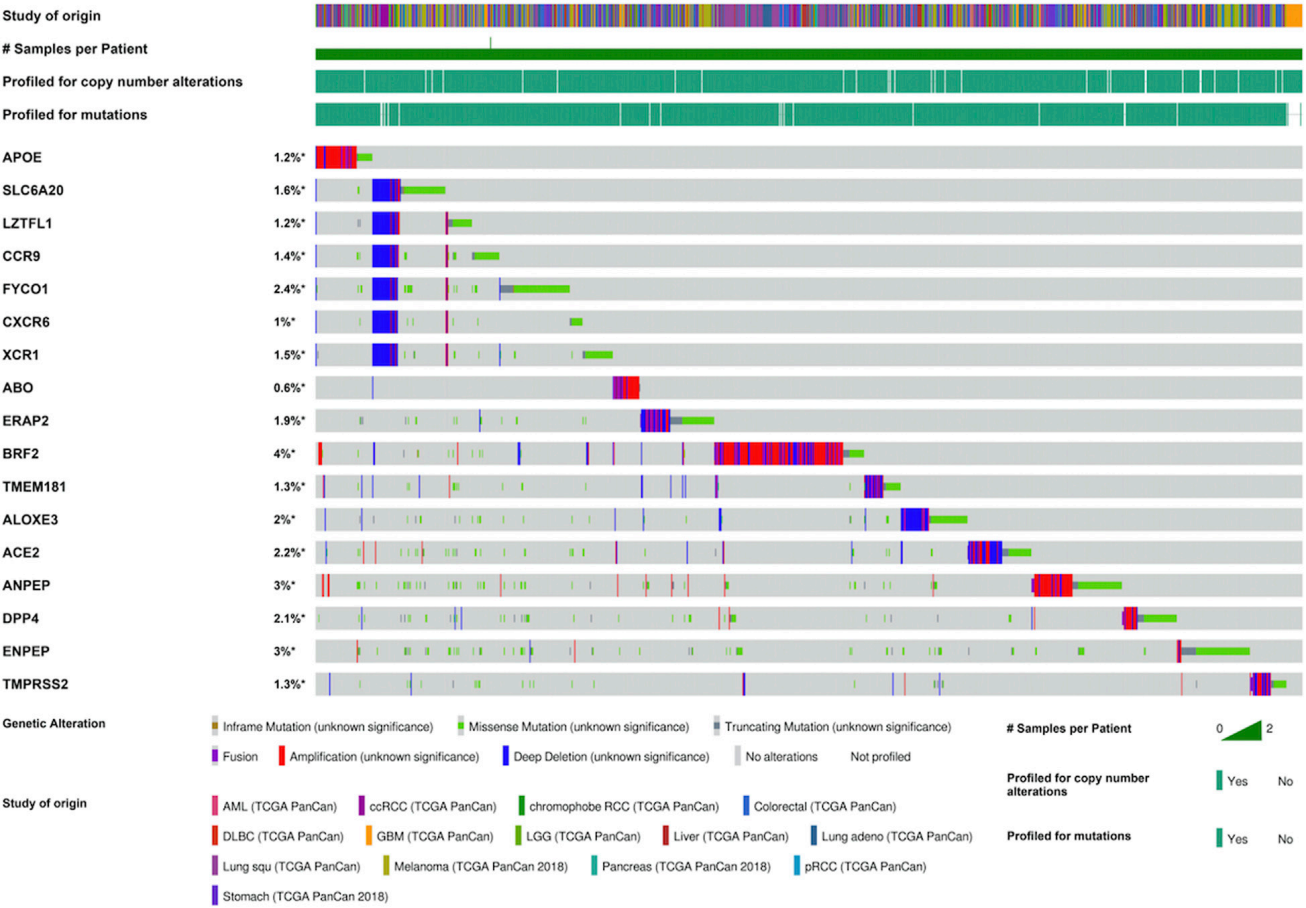
The mutation landscape of COVID-19-associated genes in different types of cancers.

**FIGURE 4:**
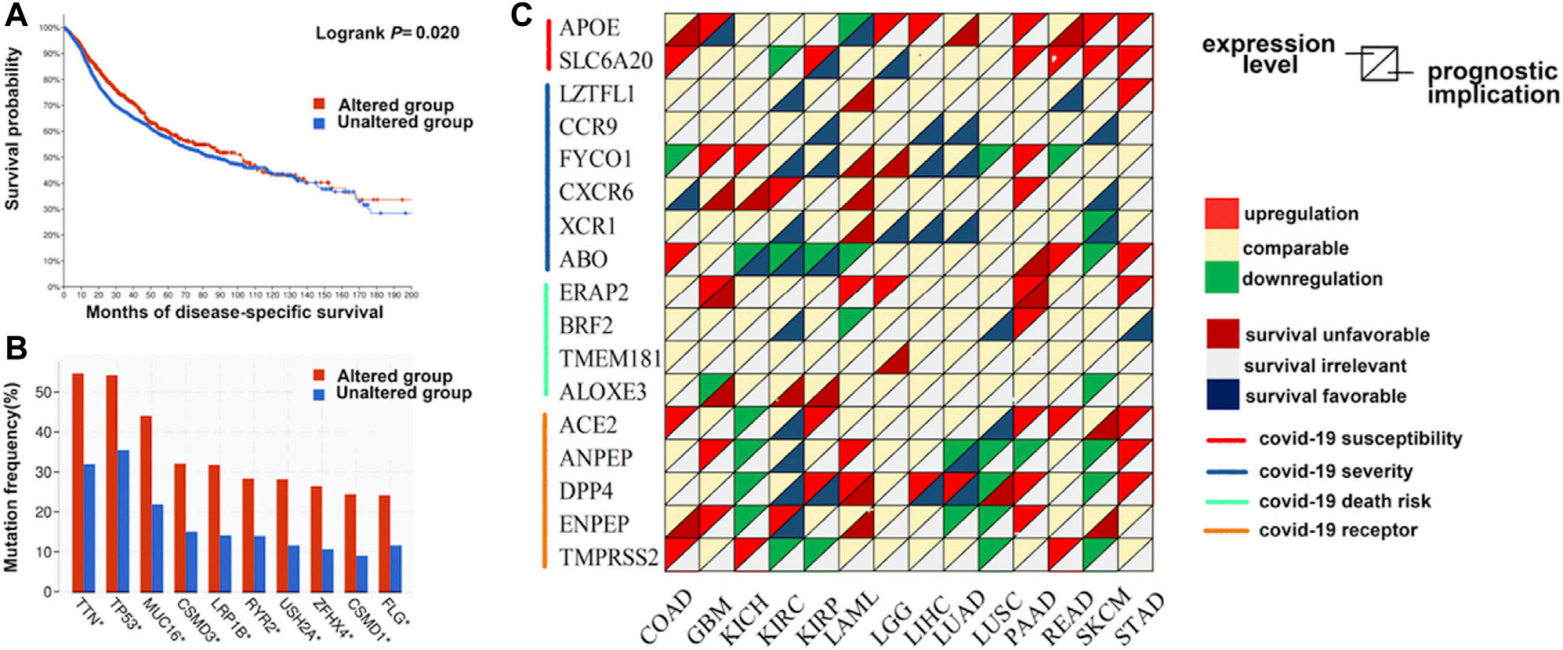
The expression pattern and prognostic implications of genes involved with COVID-19 in distinct cancers. **(A)** The disease-specific survival of patients with alterations in COVID-19-associated genes is prolonged compared with those without alterations. **(B)** Genes mutated along with the alteration of COVID-19-associated genes. **(C)** The differential expression and survival relevance of COVID-19-associated genes across distinct cancers.

Furthermore, we investigated the mutation landscape of genes involved in COVID-19 in cancers. Among these genes, 2 genes (*APOE* and *SLC6A20*) were associated with COVID-19 susceptibility, 6 genes (*LZTFL1*, *CCR9*, *FYCO1*, *CXCR6*, *XCR1*, and *ABO*) were related to COVID-19 severity, 4 genes (*ERAP2*, *BRF2*, *TMEM181*, and *ALOXE3*) were associated with COVID-19 mortality, and 5 genes (*ACE2*, *ANPEP*, *DPP4*, *ENPEP*, and *TMPRSS2*) were detected in SARS-CoV-2 receptors (Supplementary Table S8). In total, 20% (1,062/5,292) of patients harbored intratumoral mutations of at least one COVID-19-related gene. *BRF2* was the most frequently mutated gene and it mainly had amplification mutations (Figure 3). Mutations in most genes associated with COVID-19 severity featured deletions and missense mutations. Patient mutations within COVID-19-associated genes were characterized by increased disease-specific survival (*p* = 0.02) (Figure 4A). Mutual exclusivity analysis revealed broad co-occurrence among COVID-19-associated genes (Supplementary Table S9). Mutations simultaneously occurring in *LZTFL1* and *CCR9* were the most frequent across cancers. In addition, we explored whether the mutation status of other genes could be regulated by the alterations of COVID-19-associated genes. The results showed that *TTN*, *TP53,* and *MUC16* were the top 3 genes that were frequently mutated along with the alteration of COVID-19-associated genes, suggesting a potential crosstalk mechanism mutually exerted by these genes in cancer development (Figure 4B).

Next, we investigated the transcript expression pattern of these genes across distinct cancers. The differential expression of COVID-19-associated genes was universally observed between cancer and adjacent normal tissues (Figure 4C). Notably, the *APOE* transcript was upregulated in 6 cancers, while the expression of *ANPEP* was downregulated in seven cancers, and it may have oncogenic and antitumor effects. To further evaluate the correlation between the expression level of these genes and the patient prognosis, we divided patients into two groups according to the median transcript level and conducted survival analysis. Many genes were associated with patient overall and disease-free survival (Figure 4C). For example, overexpression of *DPP4* was associated with prolonged survival of patients with kidney, lung, or liver cancers. In contrast, overexpression of *APOE* was an unfavorable factor for tumors in the colon, liver, and pancreas.

## Discussion

During the COVID-19 outbreak, rational allocation of medical resources became urgent as medical and nursing resources were extremely lacking (Lee et al., 2020). Identifying vulnerable populations susceptible to COVID-19 and individuals who may suffer from severe manifestations contributes significantly to optimizing the allocation of medical resources. Questions have been raised about the biological vulnerability of patients with cancer to COVID-19, and several preliminary cross-sectional studies have also provided evidence to support this assumption. However, the causality of the association could not be confirmed due to numerous methodological biases and unmeasured confounders (Dai et al., 2020; Moujaess et al., 2020). The screening procedures for COVID-19 were more broadly and frequently performed in hospitalized patients, such as patients with cancer, than in the general population. In this context, the detection rate of COVID-19 is likely greater in patients with cancerous diseases than in the nonhospitalized population. Hence, the incidence of COVID-19 may seem to be increased in the cancerous population when the detection rate is confused with the actual incidence.

MR is an effective tool to assess the causal relationship between exposure factors and the outcome (Emdin et al., 2017). Here, we performed two-sample MR to evaluate whether a genetic predisposition to cancer is causally associated with COVID-19 susceptibility and severity based on public GWAS data. We demonstrated that a genetic predisposition to lung adenocarcinoma as opposed to lung squamous cancer is causally related to COVID-19 severity but not susceptibility (OR = 2.93, β = 1.074, se = 0.411, *p* = 0.009), suggesting that increased surveillance for severe COVID-19-associated complications should be conducted among hospitalized patients with lung adenocarcinoma. To avoid the unexpected bias from weak instruments, we calculated the *F*-statistic for SNPs used in the MR analysis. The results showed that all SNPs were qualified with the *F*-statistic larger than 10 (Supplementary Table S10). Interestingly, we found that a genetic predisposition to colorectal cancer was negatively associated with COVID-19 susceptibility (β = −0.053, se = 0.019, *p* = 0.005). However, such a small β effect may not have valuable clinical implications, and the β value mainly reflects the causal association between a genetic predisposition to colorectal cancer and COVID-19 susceptibility, while many hospitalized patients with colorectal cancer have received either cytotoxic chemotherapy or surgery, which theoretically increases the risk of succumbing to COVID-19.

We also evaluated the mutation landscape, expression pattern, and prognostic implications of genes involved with COVID-19 in distinct cancers to explore whether novel targeted treatment could be applied for patients with both cancer and COVID-19. For instance, *ERAP2* is a risk factor for COVID-19-related death and it is upregulated in lung squamous cancer but is negatively associated with the patient prognosis. Therefore, targeting *ERAP2* might be a potential treatment target to both relieve COVID-19 severity and restrain cancer progression.

To the best of our knowledge, this study is the first MR analysis to report the causal association between a genetic predisposition to lung adenocarcinoma and an increased risk for severe COVID-19, such as closer and positive surveillance to be applied to such patients in clinical practice. Specifically, for patients with lung adenocarcinoma, considering its causal association with COVID-19 severity, we suggest that chemotherapy or surgery could be postponed until they recover from COVID-19. Drugs that may exacerbate cytokine storms or lung injury should also be avoided. In addition, we explored potential molecular targets concurrently for the treatment of patients with both cancer and COVID-19, which may optimize clinical decisions precisely for such patients.

Certainly, the present study has some limitations. First, due to the inaccessibility of primary data, we cannot adjust for some key confounding factors; for example, the ethnic percentage of patients across cohorts was difficult to assess and adjust. Statistical heterogeneity and subgroup analysis were performed to minimize such unavoidable bias. Second, although our data showed no causal association between a genetic predisposition to most cancers and COVID-19 susceptibility and severity, it should not be mistaken that medical surveillance management for all of these patients could be reduced to the same level as that applied to the general population. Third, the cancer state is a binary exposure that could introduce unexpected bias, which may mitigate the causal association between the genetic predisposition to lung adenocarcinoma and increased COVID-19 severity. For patients undergoing cytotoxic chemotherapy or experienced surgery, intensified management and surveillance for COVID-19 infection and deterioration are still significant.

## Conclusion

The management of cancer patients with COVID-19 is a knotty problem whose resolution requires wisdom and a joint effort by researchers around the world. Our results highlighted the importance of strengthening medical surveillance for COVID-19 deterioration in patients with lung adenocarcinoma by showing their genetic causal association. For these patients, a delay of anticancer treatment, such as chemotherapy and surgery, should be considered.

## Data Availability

The original contributions presented in the study are included in the article/Supplementary Material. Further inquiries can be directed to the corresponding authors.

## References

[B1] Blum M. R. Tan Y. J. Ioannidis J. P. A. (2020). Use of E-Values for Addressing Confounding in Observational Studies-An Empirical Assessment of the Literature. Int. J. Epidemiol. 49, 1482–1494. 10.1093/ije/dyz261 31930286

[B2] Bowden J. Davey Smith G. Haycock P. C. Burgess S. (2016). Consistent Estimation in Mendelian Randomization with Some Invalid Instruments Using a Weighted Median Estimator. Genet. Epidemiol. 40, 304–314. 10.1002/gepi.21965 27061298 PMC4849733

[B3] Bowden J. Del Greco M F. Minelli C. Davey Smith G. Sheehan N. A. Thompson J. R. (2016). Assessing the Suitability of Summary Data for Two-Sample Mendelian Randomization Analyses Using MR-Egger Regression: the Role of the I2 Statistic. Int. J. Epidemiol. 45, 1961–1974. 10.1093/ije/dyw220 27616674 PMC5446088

[B4] Bowden J. Del Greco M. F. Minelli C. Davey Smith G. Sheehan N. Thompson J. (2017). A Framework for the Investigation of Pleiotropy in Two-Sample Summary Data Mendelian Randomization. Statist. Med. 36, 1783–1802. 10.1002/sim.7221 PMC5434863 28114746

[B5] Cerami E. Gao J. Dogrusoz U. Gross B. E. Sumer S. O. Aksoy B. A. (2012). The cBio Cancer Genomics Portal: An Open Platform for Exploring Multidimensional Cancer Genomics Data: Figure 1. Cancer Discov. 2, 401–404. 10.1158/2159-8290.cd-12-0095 22588877 PMC3956037

[B6] COVID-19 Host Genetics Initiative (2020). The COVID-19 Host Genetics Initiative, a Global Initiative to Elucidate the Role of Host Genetic Factors in Susceptibility and Severity of the SARS-CoV-2 Virus Pandemic. Eur. J. Hum. Genet. 28, 715–718. 10.1038/s41431-020-0636-6 32404885 PMC7220587

[B7] Dai M. Liu D. Liu M. Zhou F. Li G. Chen Z. (2020). Patients with Cancer Appear More Vulnerable to SARS-CoV-2: A Multicenter Study during the COVID-19 Outbreak. Cancer Discov. 10, 783–791. 10.1158/2159-8290.CD-20-0422 32345594 PMC7309152

[B8] Diao B. Wang C. Tan Y. Chen X. Liu Y. Ning L. (2020). Reduction and Functional Exhaustion of T Cells in Patients with Coronavirus Disease 2019 (COVID-19). Front. Immunol. 11, 827. 10.3389/fimmu.2020.00827 32425950 PMC7205903

[B9] Emdin C. A. Khera A. V. Kathiresan S. (2017). Mendelian Randomization. JAMA 318, 1925–1926. 10.1001/jama.2017.17219 29164242

[B10] Gao J. Aksoy B. A. Dogrusoz U. Dresdner G. Gross B. Sumer S. O. (2013). Integrative Analysis of Complex Cancer Genomics and Clinical Profiles Using the cBioPortal. Sci. Signal 6, pl1. 10.1126/scisignal.2004088 23550210 PMC4160307

[B11] Hartwig F. P. Davey Smith G. Bowden J. (2017). Robust Inference in Summary Data Mendelian Randomization via the Zero Modal Pleiotropy assumption. Int. J. Epidemiol. 46, 1985–1998. 10.1093/ije/dyx102 29040600 PMC5837715

[B12] Huang C. Wang Y. Li X. Ren L. Zhao J. Hu Y. (2020). Clinical Features of Patients Infected with 2019 Novel Coronavirus in Wuhan, China. Lancet 395, 497–506. 10.1016/s0140-6736(20)30183-5 31986264 PMC7159299

[B13] Lawlor D. A. (2016). Commentary: Two-Sample Mendelian Randomization: Opportunities and Challenges. Int. J. Epidemiol. 45, 908–915. 10.1093/ije/dyw127 27427429 PMC5005949

[B14] Lee C. C. M. Thampi S. Lewin B. Lim T. J. D. Rippin B. Wong W. H. (2020). Battling COVID‐19: Critical Care and Peri‐operative Healthcare Resource Management Strategies in a Tertiary Academic Medical centre in Singapore. Anaesthesia 75, 861–871. 10.1111/anae.15074 32267963 PMC7262214

[B15] Liang W. Guan W. Chen R. Wang W. Li J. Xu K. (2020). Cancer Patients in SARS-CoV-2 Infection: a Nationwide Analysis in China. Lancet Oncol. 21, 335–337. 10.1016/s1470-2045(20)30096-6 32066541 PMC7159000

[B16] MacArthur J. Bowler E. Cerezo M. Gil L. Hall P. Hastings E. (2017). The New NHGRI-EBI Catalog of Published Genome-wide Association Studies (GWAS Catalog). Nucleic Acids Res. 45, D896–D901. 10.1093/nar/gkw1133 27899670 PMC5210590

[B17] Mathur M. B. Ding P. Riddell C. A. VanderWeele T. J. (2018). Web Site and R Package for Computing E-Values. Epidemiology 29, e45–e47. 10.1097/ede.0000000000000864 29912013 PMC6066405

[B18] McKay J. D. Hung R. J. Han Y. Zong X. Carreras-Torres R. Christiani D. C. (2017). Large-scale Association Analysis Identifies New Lung Cancer Susceptibility Loci and Heterogeneity in Genetic Susceptibility across Histological Subtypes. Nat. Genet. 49, 1126–1132. 10.1038/ng.3892 28604730 PMC5510465

[B19] Mehta V. Goel S. Kabarriti R. Cole D. Goldfinger M. Acuna-Villaorduna A. (2020). Case Fatality Rate of Cancer Patients with COVID-19 in a New York Hospital System. Cancer Discov. 10, 935–941. 10.1158/2159-8290.cd-20-0516 32357994 PMC7334098

[B20] Moujaess E. Kourie H. R. Ghosn M. (2020). Cancer Patients and Research during COVID-19 Pandemic: A Systematic Review of Current Evidence. Crit. Rev. Oncol. Hematol. 150, 102972. 10.1016/j.critrevonc.2020.102972 32344317 PMC7174983

[B21] Sekula P. Del Greco M F. Pattaro C. Köttgen A. (2016). Mendelian Randomization as an Approach to Assess Causality Using Observational Data. J. Am. Soc. Nephrol. 27, 3253–3265. 10.1681/asn.2016010098 27486138 PMC5084898

[B22] VanderWeele T. J. Ding P. (2017). Sensitivity Analysis in Observational Research: Introducing the E-Value. Ann. Intern. Med. 167, 268–274. 10.7326/m16-2607 28693043

[B23] Zheng M. Gao Y. Wang G. Song G. Liu S. Sun D. (2020). Functional Exhaustion of Antiviral Lymphocytes in COVID-19 Patients. Cell Mol. Immunol. 17, 533–535. 10.1038/s41423-020-0402-2 32203188 PMC7091858

