# Supplementary material for: Cancer and COVID-19 Susceptibility and Severity: A Two-Sample Mendelian Randomization and Bioinformatic Analysis
Source: Front Cell Dev Biol. 2022 Jan 24;9:759257. doi: 10.3389/fcell.2021.759257 (PMC8818950; doi:10.3389/fcell.2021.759257)
Supplement: Supplementary file 1 [file DataSheet3.docx]

**Supplementary method. The essential code for MR analysis.**

*Elaborated by Dr. Yiyin Zhang*

library(TwoSampleMR)

setwd("")

exposure_dat <- read_exposure_data(filename=)

outcome_dat <- read_outcome_data(snps = exposure_dat$SNP,filename = , sep = "\t",snp_col = "rsid",beta_col = "all_inv_var_meta_beta",se_col = "all_inv_var_meta_sebeta",effect_allele_col = "ALT",other_allele_col = "REF",eaf_col = "all_meta_AF",samplesize_col = "all_meta_N")

dat <- harmonise_data(exposure_dat, outcome_dat)

res <- mr(dat)

write.table(res,file="MRresults.xls")

p1 <- mr_scatter_plot(res, dat)

mr_heterogeneity(dat)

mr_pleiotropy_test(dat)

res_single <- mr_singlesnp(dat)

res_loo <- mr_leaveoneout(dat)

res_single <- mr_singlesnp(dat)

write.table(res_single,file="MR_singleSNP.xls")

write.table(res_loo,file="MR_loo.xls")

p2 <- mr_forest_plot(res_single)

res_loo <- mr_leaveoneout(dat)

p3 <- mr_leaveoneout_plot(res_loo)

res_single <- mr_singlesnp(dat)

p4 <- mr_funnel_plot(res_single)

p1[[1]]

p2[[1]]

p3[[1]]

p4[[1]]
