# Supplementary material for: Cancer and COVID-19 Susceptibility and Severity: A Two-Sample Mendelian Randomization and Bioinformatic Analysis
Source: Front Cell Dev Biol. 2022 Jan 24;9:759257. doi: 10.3389/fcell.2021.759257 (PMC8818950; doi:10.3389/fcell.2021.759257)
Supplement: Supplementary file 2 [file DataSheet1.PDF]

Supplementary table 1. A summary of cancer-associated traits identified from GWAS catalog

Cancer types	Publication date	GWAS catalog accession	Study counts
Glioma	2012-2019	EFO_0005543	11
Squamous lung cancer	2016-2019	EFO_0000708	7
Lung adenocarcinoma	2008-2019	EFO_0000571	18
Melanoma	2008-2020	EFO_0000756	28
Lymphoid leukemia	2008-2020	EFO_0004289	56
Hepatocellular carcinoma	2012-2019	EFO_0000182	18
Colorectal carcinoma	2007-2019	EFO_0005842	76
Kidney cancer	2011-2020	MONDO_0002367	12
Gastric cancer	2010-2020	EFO_0000178	13
Pancreatic cancer	2009-2020	EFO_0002618	19

Supplementary table 2. A detailed summary of original studies reporting the selected SNPs that significantly associated with cancers.

Cancer	First author	Study accessi	Publication	Discovery samp	Replication sam	Association count
Glioma	Dahlin AM	GCST008912	2019/4/30	4543 European	\	0
	Xiao Y	GCST001470	2012/4/3	315 European	434 European	1
	Ostrom QT	GCST005932	2018/5/9	15203 European	\	14
	Ostrom QT	GCST005933	2018/5/9	13722 European	\	11
	Chen H	GCST007842	2019/2/4	2000 East Asian	5479 East Asian	5
	Kinnersley B	GCST003220	2015/10/1	9218 European	3213 European	7
	Ostrom QT	GCST006480	2018/8/27	15094 NR	\	20
	Ostrom QT	GCST005931	2018/5/9	18723 European	\	17
	Melin BS	GCST004349	2017/3/27	24381 European	\	13
	Melin BS	GCST004347	2017/3/27	30659 European	\	18
	Melin BS	GCST004348	2017/3/27	24009 European	\	19
Pancreatic cancer	Willis JA	GCST001556	2012/6/4	252 European	10 East Asian21	0
	Diergaarde B	GCST000686	2010/5/17	190 European	142 European	0
	Innocenti F	GCST001346	2011/12/5	294 European	\	3
	Low SK	GCST000745	2010/7/29	6200 East Asian	\	17
	Kiyotani K	GCST001390	2012/1/29	79 East Asian	95 East Asian	2
	Rashkin SR	GCST900118:	2020/9/4	411013 Europea	\	12
	Chang J	GCST009895	2018/9/11	4851 East Asian	6839 East Asian	3
	Innocenti F	GCST007643	2019/3/18	294 European	\	6
	Wu C	GCST001749	2012/11/24	642 European	363 East Asian	7
	Wu C	GCST001350	2011/12/11	2972 East Asian	5480 East Asian	11
	Lin Y	GCST010616	2020/6/24	34631 East Asia	10822 East Asian	0
	Zhang M	GCST003758	2016/8/1	13952 European	13631 NR	3
	Tang H	GCST004485	2017/5/3	868 European	820 European	61
	Amundadotti	GCST000456	2009/8/2	3576 European	4247 European	1
	Petersen GM	GCST000574	2010/1/24	126 East Asian7	\	3
	Childs EJ	GCST002991	2015/6/22	7046 NR636 NI	6492 NR, Europ	17
	Walsh N	GCST007235	2018/12/12	21536 European	\	9
	Klein AP	GCST005434	2018/2/8	21536 European	7489 NR	32
	Wolpin BM	GCST002553	2014/8/3	6785 European	15295 European	10
Squamous lung cance	Zanetti KA	GCST003777	2016/5/13	4001 African A	1329 African An	0
	Dai J	GCST008835	2019/7/17	17188 East Asia	\	3
	Byun J	GCST006088	2018/6/19	31930 European	\	68
	Li Y	GCST005911	2017/10/20	4529 European	\	0
	Fehringer G	GCST003587	2016/4/20	123671 Europea	\	17
	McKay JD	GCST004750	2017/6/12	63053 European	\	101
	Fehringer G	GCST003588	2016/4/20	123671 Europea	\	26
Lung adenocarcinoma	Galvan A	GCST000223	2008/8/26	1004 European	\	0
	Miki D	GCST000810	2010/9/26	2904 East Asian	10242 East Asian	2
	Frullanti E	GCST000947	2011/1/17	600 European	317 European	0
	Shiraishi K	GCST001609	2012/7/15	7028 East Asian	12536 East Asian	4
	Zanetti KA	GCST003776	2016/5/13	4340 African A	1448 African An	1
	Chang IS	GCST003794	2016/9/26	128 East Asian	70 East Asian15:	0
	Byun J	GCST006087	2018/6/19	32338 European	\	36
	Landi MT	GCST000506	2009/10/15	11587 European	21379 European	5
	Hsiung CA	GCST000761	2010/8/5	1169 East Asian	4699 East Asian	1
	Hung RJ	GCST008375	2019/4/19	7192 European	1612 European	1
	Shiraishi K	GCST003605	2016/8/9	5030 East Asian	13301 East Asian	6
	Li Y	GCST005909	2017/10/20	7015 European	\	0
	Dai J	GCST008836	2019/7/17	20846 European	\	19
	Wang Z	GCST003326	2016/1/4	13154 East Asia	12924 East Asian	5

Wang Y	GCST002466	2014/6/1	27209 Europear	48541 European	5
McKay JD	GCST004744	2017/6/12	66756 Europear	\	79
Fehringer G	GCST003587	2016/4/20	123671 Europea	\	17
Fehringer G	GCST003588	2016/4/20	123671 Europea	\	26

Melanoma

Thomsen H	GCST009075	2019/10/11	571 European	\	4
Thomsen H	GCST009078	2019/10/11	5637 European	\	6
Papakostas T	GCST005589	2018/3/1	126 NR	\	0
Thomsen H	GCST009076	2019/10/11	5484 European	\	4
Mobuchon L	GCST004661	2017/3/10	660 European	460 European	2
Thomsen H	GCST009079	2019/10/11	5616 European	\	5
Thomsen H	GCST009077	2019/10/11	5452 European	\	3
Thomsen H	GCST009074	2019/10/11	5529 European	\	4
Thomsen H	GCST009073	2019/10/11	5454 European	\	3
Thomsen H	GCST009071	2019/10/11	5789 European	\	10
Teerlink C	GCST001129	2011/6/26	2306 European	\	1
Kulkarni D	GCST003456	2016/3/29	579 European	\	0
Amos CI	GCST001245	2011/9/22	2830 European	29807 European,	1
Song F	GCST002514	2014/6/30	6122 European	18416 European	6
Bishop DT	GCST000437	2009/7/5	5456 European	4179 European	5
Brown KM	GCST000198	2008/5/18	1728 European	2481 European	1
Macgregor S	GCST001266	2011/10/9	6555 European	20337 European	2
Rashkin SR	GCST900118(	2020/9/4	417127 Europea	\	38
Ransohoff K.	GCST004142	2017/2/9	291407 Europea	2830 European	28
Vaysse A	GCST003639	2016/6/27	966 European	1546 European	0
Barrett JH	GCST001267	2011/10/9	10422 Europear	13000 European	10
Iles MM	GCST001886	2013/3/3	4919 European	67980 European	1
Duffy DL	GCST007505	2018/11/14	88583 Europear	\	30
Avitabile M	GCST010152	2019/9/7	42380 Europear	6016 European	16
Law MH	GCST003061	2015/8/3	36077 Europear	4746 NR1576 Eu	16
Landi MT	GCST010302	2020/4/27	352662 Europea	\	48
Landi MT	GCST010304	2020/4/27	411948 Europea	\	76
Landi MT	GCST010303	2020/4/27	424878 Europea	\	66

Qian M	GCST010756	2019/4/2	126 Hispanic or	5518 African An	0
Rashkin SR	GCST900118:	2020/9/4	411202 Europea	\	24
Vijayakrishna	GCST009637	2019/11/25	18035 Europear	3883 NR	1
Vijayakrishna	GCST009636	2019/11/25	17602 Europear	3826 NR	1
Vijayakrishna	GCST009638	2019/11/25	21987 Europear	5698 NR	10
Vijayakrishna	GCST005832	2018/4/9	17051 Europear	\	11
Han S	GCST000608	2010/2/25	93 East Asian	\	0
Horinouchi M	GCST000749	2010/8/1	8 East Asian	16 East Asian	0
Tulstrup M	GCST007149	2018/9/10	454 European	\	1
Tulstrup M	GCST007150	2018/9/10	443 European	\	1
Tulstrup M	GCST007151	2018/9/10	443 European	136 European	0
Tulstrup M	GCST007148	2018/9/10	452 European	\	1
Liu C	GCST003609	2016/8/26	407 European11	\	1
Ellinghaus E	GCST001320	2011/11/11	893 European	4338 European	31
Diouf B	GCST002792	2015/2/24	209 European44	\	5
Fernandez C/	GCST002915	2015/5/18	3126 European,	182 European, H	8
Qian M	GCST007276	2018/12/3	1521 Hispanic c	585 Hispanic or	0
Archer NP	GCST007727	2017/8/17	203 Hispanic or	\	2
Liu Y	GCST004250	2017/1/16	60 Hispanic or I	\	54
Mateos MK	GCST010463	2020/5/19	1573 European	\	26
Berndt SI	GCST003468	2016/3/9	10767 Europear	7488 NR, Europe	31
Law PJ	GCST004146	2017/2/6	17691 Europear	6107 European	37
Law PJ	GCST004099	2017/1/23	14421 Europear	\	20
Clay-Gilmou	GCST005965	2017/9/8	3220 European	\	3

	Clay-Gilmou	GCST005966	2017/9/8	3437 European	\	3
	Clay-Gilmou	GCST005967	2017/9/8	3360 European	\	3
	Clay-Gilmou	GCST005968	2017/9/8	3280 European	\	1
	Clay-Gilmou	GCST005969	2017/9/8	3161 European	\	1
	Clay-Gilmou	GCST005970	2017/9/8	3187 European	\	1
	Clay-Gilmou	GCST005971	2017/9/8	3179 European	\	1
	Clay-Gilmou	GCST005972	2017/9/8	3473 European	\	3
**Lymphoid leukemia**	Vijayakrishna	GCST003798	2016/10/3	8882 European	2891 European3	2
	Migliorini G	GCST002158	2013/8/30	6381 European	2937 European	6
	Slager SL	GCST000906	2010/12/3	703 European	1217 European	4
	Di Bernardo	GCST000224	2008/8/31	1943 European	2701 European	6
	Slager SL	GCST001570	2012/6/13	4866 European	2894 European	8
	Inaba H	GCST005214	2017/12/19	13 NR235 Euro	\	4
	Wiemels JL	GCST005315	2018/1/18	3972 African A	3583 European1(	9
	Perez-Andreu	GCST002713	2014/12/2	1480 European1	535 Hispanic or	1
	Papaemmanu	GCST000463	2009/8/16	3305 European	\	3
	Yang JJ	GCST000323	2009/1/28	53 African unsp	\	12
	Treviño LR	GCST000464	2009/8/16	19275 European	\	14
	Yang JJ	GCST001693	2012/9/24	1268 African A	1267 African An	10
	Xu H	GCST001912	2013/3/19	1452 African A	3175 European1	3
	Evans TJ	GCST002657	2014/10/13	1550 European	1983 European	2
	Perez-Andreu	GCST002236	2013/10/20	4344 African A	920 Hispanic or	0
	Liu C	GCST003500	2016/4/25	213 NR, Europe	\	0
	Liu C	GCST003501	2016/4/25	99 Asian unspe	\	11
	Højfeldt SG	GCST007540	2018/11/18	831 European	\	11
	Højfeldt SG	GCST007539	2018/11/18	803 European	\	0
	Højfeldt SG	GCST007541	2018/11/18	878 European	\	0
	Speedy HE	GCST002299	2013/12/1	6938 European	4295 European	19
	Din L	GCST008721	2019/8/13	36793 European	\	7
	Din L	GCST008724	2019/8/13	17645 European	\	1
	Din L	GCST008727	2019/8/13	32855 European	\	4
	Berndt SI	GCST002073	2013/6/16	8400 European	8027 European	21
**Hepatocellular carcinoma**	Chan KY	GCST001348	2011/12/8	192 East Asian	1228 East Asian	0
	Lin YY	GCST004736	2017/6/29	278 East Asian	\	4
	Kumar V	GCST001041	2011/4/17	3611 East Asian	3269 East Asian	2
	Clifford RJ	GCST000902	2010/12/1	451 East Asian	622 East Asian	3
	Yang C	GCST008104	2018/12/18	408 East Asian	\	0
	Zhang H	GCST000752	2010/8/1	707 East Asian	3869 East Asian	1
	Yang CK	GCST007514	2017/7/13	195 East Asian	97 East Asian	0
	Qu LS	GCST003189	2015/11/23	100 East Asian	560 East Asian	2
	Kawaguchi T	GCST005309	2018/1/31	7730 East Asian	\	3
	Wei J	GCST007731	2019/4/4	367 East Asian	758 East Asian	5
	Prasad G	GCST007650	2019/3/1	367 East Asian	758 East Asian	5
	Li Y	GCST005570	2018/2/15	1274 East Asian	6340 East Asian	2
	Matsuura K	GCST004160	2017/2/2	457 East Asian	340 East Asian	1
	Li S	GCST001603	2012/7/12	3003 East Asian	9156 East Asian	3
	Jiang DK	GCST001775	2012/12/16	2514 East Asian	9285 East Asian	2
	Lee MH	GCST006037	2017/9/16	1251 East Asian	1098 East Asian	1
	Sawai H	GCST005746	2018/5/21	989 East Asian	1048 East Asian	7
	Alberts R	GCST005858	2017/8/4	3006 European	\	0
	Penney KL	GCST010716	2019/12/4	107 NR4319 Eu	\	1
	Penney KL	GCST010717	2019/12/4	107 NR4319 Eu	\	1
	Penney ME	GCST006411	2018/6/13	505 European	\	17
	Dorani F	GCST008977	2018/10/29	944 European	\	0
	Takahashi Y	GCST004840	2017/9/4	1050 East Asian	2798 East Asian	0
	Hong SN	GCST002099	2013/7/22	105 East Asian	379 East Asian	0

**Colorectal carcinoma**	Spain SL	GCST000472	2009/9/1	1850 European	2649 European	0
	Takahashi Y	GCST004839	2017/9/4	1050 East Asian	3373 East Asian	1
	Wang M	GCST003494	2016/5/5	2329 East Asian	2122 European1	4
	Lascorz J	GCST000722	2010/7/7	1634 European	13074 European	0
	Schmit SL	GCST002528	2014/7/14	983 Other2976	1962 Other	1
	Cui R	GCST000948	2011/1/17	3481 East Asian	7782 East Asian	2
	Fernandez-Ro	GCST001832	2013/1/26	1355 European	3216 European	3
	Figueiredo JC	GCST000989	2011/2/25	2190 European	1682 European	0
	Siegert S	GCST001731	2012/11/2	314 European	1261 European	0
	Tomlinson I	GCST000053	2007/7/8	1890 European	12580 European	1
	Zhang B	GCST002340	2014/1/21	4415 East Asian	14764 East Asian	1
	Schmit SL	GCST003420	2016/4/18	5941 Hispanic o	\	7
	Hofer P	GCST005148	2017/10/9	2469 European	\	6
	Hofer P	GCST005152	2017/10/9	1833 European	\	13
	Baas J	GCST007259	2018/12/17	282 NR	\	8
	Zhou W	GCST008372	2018/8/13	387318 Europea	\	3
	Tanskanen T	GCST004895	2017/9/28	15783 European	24003 European	5
	Gong J	GCST003873	2016/10/10	16823 European	\	2
	Gong J	GCST003874	2016/10/10	22601 European	\	0
	Garcia-Alben	GCST003385	2016/1/14	3384 European	\	0
	Garcia-Alben	GCST003386	2016/1/14	1283 European	\	1
	Garcia-Alben	GCST003387	2016/1/14	1606 European	\	0
	Du M	GCST002596	2014/9/5	18509 European	\	3
	Whiffin N	GCST002411	2014/4/15	13443 European	29974 European	9
	Zanke BW	GCST000052	2007/7/8	2194 NR	2194 NR10472 E	1
	Peters U	GCST001161	2011/7/15	6322 European	17262 European	5
	Houlston RS	GCST000843	2010/10/24	30420 European	\	5
	Houlston RS	GCST000270	2008/11/16	3831 European	37210 European	4
	Tenesa A	GCST000168	2008/3/30	1983 European	3560 NR20688 E	3
	Figueiredo JC	GCST002415	2014/4/17	18404 European	\	13
	Phipps AI	GCST003229	2015/11/19	3494 European	3764 European	1
	Phipps AI	GCST003231	2015/11/19	2843 European	3205 European	1
	Phipps AI	GCST003230	2015/11/19	462 European	435 European	4
	Tanikawa C	GCST005591	2018/2/17	33870 East Asia	11046 East Asian	30
	Hofer P	GCST005149	2017/10/9	5272 European	\	42
	Hofer P	GCST005150	2017/10/9	5908 European	\	42
	Alberts R	GCST005856	2017/8/4	2949 European	\	1
	Dunlop MG	GCST001544	2012/5/27	17780 European	37170 European	3
	Zhang B	GCST002454	2014/5/18	8270 East Asian	38638 East Asian	20
	Tomlinson IP	GCST000169	2008/3/30	1849 European	33951 European	5
	Peters U	GCST001787	2012/12/21	27809 European	1867 European7	15
	Jiao S	GCST001794	2012/12/26	18938 European	5185 European	7
	Schumacher I	GCST003017	2015/7/7	37955 European	14694 East Asian	22
	Cheng TH	GCST003209	2015/12/1	21333 European	\	16
	Cheng TH	GCST003208	2015/12/1	21333 European	31179 European	13
	Zeng C	GCST003799	2016/3/8	21096 East Asia	32599 East Asian	30
	Lu Y	GCST007552	2018/12/6	70506 East Asia	\	40
	Lu Y	GCST009869	2019/12/11	72272 East Asia	125478 European	70
	Wang H	GCST004169	2017/3/13	5146 African A	1434 African An	0
	Pander J	GCST003058	2015/7/29	520 European	\	3
	Pander J	GCST003057	2015/7/29	520 European	\	1
	Schmit SL	GCST006131	2018/6/16	6597 African A	5941 Hispanic or	28
	Fehringer G	GCST003587	2016/4/20	123671 Europea	\	17
	Rashkin SR	GCST900118	2020/9/4	414143 Europea	\	13
	Xu W	GCST002821	2015/3/19	171 European	\	18
	Rashkin SR	GCST900118	2020/9/4	412441 Europea	\	12
	Penney ME	GCST007434	2019/2/9	379 European	\	26
	Huyghe JR	GCST007856	2018/12/3	120184 Europea	\	121

	Fehringer G	GCST003588	2016/4/20	123671 Europea	\	26
	Real LM	GCST002513	2014/6/30	1281 European	3354 European	0
	Real LM	GCST002512	2014/6/30	1281 European	3354 European	0
	Broderick P	GCST000113	2007/10/14	1890 European	13475 European	1
	Xu W	GCST002820	2015/3/19	431 European	\	12
	Wang H	GCST004168	2017/3/13	6597 African A	5941 Hispanic o	2
	Wang H	GCST002561	2014/8/8	6596 African A	35034 European	1
	Nan H	GCST002816	2015/3/17	17187 Europear	\	2
	Jia WH	GCST001792	2012/12/23	7847 East Asiar	26060 European	7
	Al-Tassan N	GCST002919	2015/5/20	17556 Europear	\	19
	Pande M	GCST006035	2018/4/26	2754 European	\	18
	Law PJ	GCST007992	2019/5/14	92967 Europear	\	87
Kidney cancer	Turnbull C	GCST001500	2012/4/29	2636 European	5339 European	7
	Rashkin SR	GCST900118	2020/9/4	411688 Europea	\	10
	Purdue MP	GCST002272	2013/11/12	630 African Am	683 African Ame	0
	Henrion MY	GCST002835	2015/3/31	10871 Europear	10114 European	3
	Gudmundsso	GCST002273	2013/11/13	69230 Europear	4678 European	1
	Henrion M	GCST001750	2012/11/25	10781 Europear	12525 European	2
	Wu X	GCST001283	2011/10/18	2410 European	12277 European	3
	Purdue MP	GCST000907	2010/12/5	12277 Europear	7116 European	4
	Laskar RS	GCST008225	2019/6/23	5087 European	2974 European	3
	Laskar RS	GCST008224	2019/6/23	13230 Europear	11087 European	1
	Laskar RS	GCST008226	2019/6/23	8143 European	8113 European	8
	Scelo G	GCST004710	2017/6/9	31190 Europear	9483 European	26
Gastric cancer	Helgason H	GCST002990	2015/6/22	204576 Europea	\	7
	Hu N	GCST003009	2015/6/30	3897 East Asiar	11286 East Asia	1
	Shi Y	GCST001300	2011/10/30	3279 East Asiar	6897 East Asian	2
	Abnet CC	GCST000777	2010/8/22	5623 East Asiar	\	4
	Du M	GCST010462	2020/5/20	3725 East Asiar	12038 East Asia	2
	Rashkin SR	GCST900118	2020/9/4	411441 Europea	\	11
	Hu N	GCST003010	2015/6/30	5058 East Asiar	14956 East Asia	4
	Hu N	GCST003007	2015/6/30	3735 East Asiar	11211 East Asia	2
	Helgason H	GCST002992	2015/6/22	208152 Europea	\	8
	Tanikawa C	GCST006707	2018/10/3	33349 East Asia	17062 East Asia	6
	Yan C	GCST008646	2019/8/5	9197 East Asiar	15358 East Asia	6
	Wang Z	GCST003218	2015/12/23	7001 East Asiar	8201 East Asian	6
	Jin G	GCST001718	2012/10/25	9374 East Asiar	20437 East Asia	7

Note: "NR" refers to "not reported"; "\" refers to "not available".

By COVID19-hg GWAS meta-analysis round 4

Supplementary table 3. A summary of GWAS study for COVID-19 used in the present study

**Phenotype**	**Covid-19**	**Laboratory/self-reported negative**
**Total cases**	24057	218062
**Contributing studies**		
UKBB_AFR	68	268
UKBB_CSA	66	350
UKBB_EUR	1310	13646
DECODE_EUR	1897	29014
EstBB_EUR	313	12019
Genomicsengland100kg	218	1617
GNH_SAS	114	256
Lifelines_EUR	358	1253
MGI_EUR	122	508
MVP_AFR	1217	9204
MVP_EUR	1520	24135
MVP_HIS	510	3325
NTR_EUR	145	117
PHBB_AFR	60	375
PHBB_EUR	151	3118
PHBB_HIS	66	276
PMBB_AFR	166	934
BQC19_EUR	206	327
INTERVAL_EUR	161	1119
23ANDME_AFR	506	3110
23ANDME_EUR	9913	85072
23ANDME_HIS	2553	13086
Ancestry_EUR	2417	14933
	**Very severe respirator**	**Not hospitalized covid**
**Total cases**	269	688
**Contributing studies**		
BoSCO_EUR	59	262
FinnGen_FIN	54	224
SPGRX_EUR	101	51
BQC19_EUR	55	151

Supplementary table 4. The effect value of each SNP used in the MR analysis.

Glioma & covid susceptibility				Glioma & covid severity			
**SNP**	**beta**	**se**	**P_value**	**SNP**	**beta**	**se**	**P_value**
rs1006969	-0.00105	0.035928	0.976702	rs10069690	-0.52978	0.337796	0.1168
rs1013103	-0.08191	0.098184	0.40416	rs10131032	0.018622	0.889652	0.9833
rs1084289	0.067754	0.037981	0.074443	rs10842893	-0.13053	0.333943	0.695893
rs1123325	0.009135	0.112226	0.935123	rs11233250	-0.70777	0.980608	0.470436
rs1159977	0.062685	0.114216	0.583125	rs11599775	-0.07369	1.016844	0.942229
rs1275255	-0.0009	0.118767	0.993944	rs12752552	0.108649	1.053	0.91782
rs2297440	0.085509	0.048194	0.076019	rs2297440	0.51548	0.459415	0.261847
rs3751667	0.096644	0.116648	0.407383	rs3751667	-1.1721	1.088848	0.281721
rs3851634	-0.17026	0.093209	0.067754	rs3851634	0.462873	0.733909	0.52824
rs4252707	0.160463	0.114663	0.161685	rs4252707	-1.87119	1.015847	0.065475
rs5570585	-0.03874	0.031438	0.217846	rs55705857	-0.2563	0.292531	0.380948
rs634537	-0.04951	0.053194	0.352024	rs634537	0.806929	0.480511	0.093091
rs7572263	0.036809	0.098096	0.707485	rs7572263	1.530044	0.933461	0.101191
rs7837822	-0.03747	0.075202	0.618291	rs78378222	1.523397	0.694831	**0.028345**
All - Invers	0.004869	0.017238	0.777584	All - Inverse va	-0.01187	0.178109	0.946854
All - MR E	-0.00988	0.031692	0.760625	All - MR Egge	-0.05278	0.331306	0.876082
**quamous lung cancer & covid susceptibilit**				**Squamous lung cancer & covid severity**			
rs1138550	0.037739	0.159368	0.812811	rs12686364	0.562161	0.609008	0.355967
rs1157181	-0.09813	0.139184	0.480767	rs13191296	-1.92585	1.629887	0.237369
rs1268636	0.004249	0.056193	0.939728	rs13218875	-2.88476	1.653821	0.081107
rs1319129	0.085788	0.135658	0.527138	rs13220495	-2.41897	2.376235	0.308686
rs1321887	0.13724	0.141721	0.332857	rs148696809	-2.64526	1.495621	0.076949
rs1322049	0.126143	0.173951	0.468353	rs149949	-1.23727	1.570712	0.430864
rs1486968	0.093194	0.135053	0.490161	rs34107459	-1.31723	1.622223	0.416796
rs149949	0.09497	0.124326	0.444941	rs34878803	-2.83314	1.554907	0.068445
rs3410745	0.178562	0.136465	0.190708	rs35768595	-2.4024	2.136884	0.260905
rs3487880	0.031677	0.129365	0.80656	rs36109883	-1.39912	1.513862	0.35538
rs3576859	0.095514	0.143933	0.506946	rs56404467	-0.69757	1.223183	0.56848
rs3610988	0.091074	0.123928	0.462405	rs8040868	2.006207	0.591959	**0.000701**
rs5640446	-0.21652	0.155928	0.164948				
rs7160339	-0.02332	0.04954	0.63777				
rs8040868	-0.03278	0.062239	0.598421				
All - Invers	0.012411	0.025016	0.619797	All - Inverse va	0.007796	0.519156	0.988019
All - MR E	-0.04594	0.046823	0.344381	All - MR Egge	0.948439	1.048799	0.387098
**ung adenocarcinoma & covid susceptibilit**				**Lung adenocarcinoma & covid severity**			
rs1159171	0.036759	0.14611	0.801364	rs11591710	0.203995	1.489262	0.891049
rs1163203	-0.02618	0.180548	0.884729	rs11632038	1.007553	1.384078	0.466638
rs1163937	-0.03554	0.094833	0.707818	rs11639372	1.706989	0.917962	0.06295
rs1703856	-0.39141	0.272506	0.150909	rs17038564	-2.71827	3.610425	0.451514
rs1740042	0.072871	0.158648	0.646	rs17400427	0.815891	1.400003	0.560043
rs2004038	-0.17395	0.117606	0.139122	rs2004038	2.476617	1.254452	**0.048352**
rs2495239	-0.0907	0.176841	0.608017	rs2495239	1.383302	1.617733	0.392503
rs2840831	-0.03162	0.188373	0.866701	rs28408315	1.017338	1.488866	0.494419
rs2851644	0.058016	0.192217	0.762787	rs28516445	0.696035	1.519729	0.646953
rs3451743	0.181222	0.19381	0.349765	rs34517439	3.324651	1.783637	0.062325
rs4324798	0.303665	0.190924	0.111722	rs4324798	-4.62262	2.280892	**0.042696**
rs6256077	-0.04495	0.15002	0.764438	rs62560775	0.017083	1.43473	0.9905
rs7086803	0.262648	0.187289	0.160805	rs7086803	4.016447	2.575727	0.118915
rs7169304	-0.05177	0.15539	0.739035	rs7169304	1.964462	1.395327	0.159165
rs7741164	0.449592	0.244548	0.065995	rs7741164	-6.28272	3.76762	0.095404
rs885518	0.077465	0.148758	0.602542	rs885518	0.622142	1.482983	0.674835
All - Invers	0.011576	0.039785	0.771074	All - Inverse va	1.073719	0.41089	**0.008971**
All - MR E	0.133031	0.253712	0.608245	All - MR Egge	1.193813	2.567002	0.649039
**Melanoma & covid susceptibility**				**Melanoma & covid severity**			
rs1159171	0.04968	0.197469	0.801364	rs11591710	0.2757	2.012746	0.891049

rs1301696	0.094766	0.121127	0.433997	rs13016963	1.460448	1.118003	0.19145
rs1393350	-0.03374	0.118156	0.775249	rs1393350	-0.52029	1.112903	0.640139
rs1695300	-0.17349	0.153524	0.258461	rs16953002	0.530569	1.278264	0.678091
rs1878436	-0.11829	0.224016	0.597463	rs4436359	-2.42633	1.856426	0.191216
rs4436359	-0.10121	0.230648	0.660794	rs45430	-0.09589	1.360206	0.9438
rs6059655	0.093923	0.095118	0.32343	rs6059655	1.022002	1.062302	0.336018
rs6238942	0.068704	0.112481	0.54133	rs62389423	-1.45427	1.246796	0.24345
rs9745545	0.016114	0.164072	0.921763	rs9745545	0.902545	1.2844	0.482244
All - Inver	0.021353	0.045966	0.64226	All - Inverse va	0.166123	0.42904	0.698611
All - MR E	0.077096	0.125467	0.558342	All - MR Egge	0.667143	1.430066	0.65502
**Lymphoid leukemia & covid susceptibility**				**Lymphoid leukemia & covid severity**			
rs1082193	0.001479	0.027889	0.957695	rs10821936	-0.12859	0.275134	0.64024
rs1108384	0.025386	0.063834	0.690857	rs11083846	1.178223	0.577165	**0.041212**
rs1136505	0.048531	0.05836	0.405649	rs113650570	-0.76926	0.613284	0.209725
rs1197826	0.007273	0.033232	0.826776	rs11978267	-0.20231	0.319537	0.526637
rs1198037	0.011218	0.021026	0.593675	rs11980379	-0.12922	0.198713	0.515503
rs1277930	-0.08339	0.081176	0.30427	rs12779301	0.902133	0.77626	0.245173
rs1301579	0.26291	0.104938	0.012231	rs13015798	-0.00274	0.866475	0.99748
rs1340181	-0.02697	0.056809	0.634985	rs13401811	0.407026	0.529178	0.441794
rs1713380	0.014733	0.035273	0.676173	rs17133805	-0.13982	0.334242	0.675719
rs1748186	0.039324	0.056261	0.484577	rs17481869	0.115192	0.465458	0.804536
rs1748346	0.035016	0.061126	0.566743	rs17483466	-0.02615	0.540474	0.961405
rs210142	0.058825	0.051425	0.252663	rs210142	-0.54727	0.538588	0.309576
rs2296624	-0.02096	0.079518	0.792132	rs2296624	0.62946	0.745171	0.398267
rs2953196	-0.02962	0.072647	0.683442	rs2953196	0.178885	0.681267	0.792877
rs3583778	-0.08163	0.083952	0.330861	rs35837782	0.950371	0.7964	0.232739
rs3592364	0.010662	0.040821	0.793944	rs35923643	-0.15126	0.375678	0.687227
rs4127147	-0.07482	0.113076	0.508152	rs41271473	0.415813	1.08144	0.700608
rs4245595	-0.00473	0.03562	0.894334	rs4245595	-0.15474	0.331	0.640151
rs4982731	0.005155	0.055629	0.926162	rs4982731	-0.09858	0.497553	0.842948
rs4987856	0.115476	0.084409	0.171293	rs4987856	-0.12944	0.652745	0.842805
rs5721427	0.040339	0.135545	0.766006	rs57214277	-0.67568	1.248756	0.588451
rs5805567	0.025206	0.059765	0.673207	rs58055674	0.188203	0.514668	0.714605
rs674313	-0.02996	0.03385	0.376077	rs674313	-1.12062	0.35001	**0.001366**
rs7159710	0.007489	0.128398	0.953489	rs71597109	0.630259	1.083668	0.560838
rs7176508	0.013676	0.050395	0.786111	rs7176508	0.51936	0.482798	0.282049
rs7274268	-0.02023	0.074227	0.7852	rs72742684	0.685763	0.735194	0.350941
rs735665	0.01706	0.055124	0.756959	rs735665	-0.59153	0.552072	0.28396
rs7577761	0.073341	0.103595	0.47897	rs75777619	0.232398	0.953911	0.80752
All - Inver	0.008753	0.009131	0.33777	All - Inverse va	-0.0862	0.08641	0.318465
All - MR E	0.010061	0.02115	0.638269	All - MR Egge	-0.50316	0.198938	**0.017836**
**patocellular carcinoma & covid susceptibil**				**Hepatocellular carcinoma & covid severity**			
rs1740196	-0.01179	0.03506	0.736579	rs17401966	-0.05884	0.324543	0.856121
rs7574865	0.112186	0.098652	0.255456	rs7574865	-2.112	0.986463	**0.032276**
rs9275319	0.165697	0.055979	**0.003076**	rs9275319	0.734297	0.530649	0.166428
All - Inver	0.044366	0.055956	0.427853	All - Inverse va	-0.00862	0.481684	0.985719
All - MR E	-0.06038	0.203056	0.816008	All - MR Egge	1.383728	1.446895	0.514204
**Colorectal carcinoma& covid susceptibility**				**Colorectal carcinoma & covid severity**			
rs1004939	-0.48697	0.315709	0.122963	rs10049390	1.087184	2.769573	0.694655
rs1035209	0.002747	0.178331	0.987711	rs1035209	-0.01	1.714659	0.995347
rs1041121	-0.13235	0.158448	0.403542	rs10411210	-0.07637	1.597073	0.961859
rs1050686	-0.6474	0.494816	0.190748	rs10506868	1.407405	4.618185	0.760554
rs1079566	-0.08343	0.124133	0.501493	rs10774214	-0.07086	1.086941	0.948018
rs1082190	-0.17795	0.282195	0.528313	rs10795668	0.191404	1.072753	0.858391
rs1084943	0.120791	0.172436	0.483618	rs10821907	-2.40342	2.324683	0.301196
rs1084943	-0.06904	0.289331	0.811405	rs10849432	0.72595	1.781753	0.683688
rs1084943	0.001733	0.223818	0.993822	rs10849433	-2.37708	2.748464	0.387106
rs1098062	0.259568	0.293266	0.376107	rs10849438	-2.13653	1.892636	0.258955

rs1106443	0.582915	0.365644	0.110888	rs10980628	-1.01691	2.658345	0.702064
rs1108778	-0.25714	0.292721	0.379695	rs11064437	1.653685	8.992428	0.854094
rs1119016	-0.11182	0.215218	0.603366	rs11087784	-1.91964	2.382287	0.42036
rs1135695	-0.06925	0.220092	0.753024	rs11190164	-0.50341	1.997385	0.801013
rs1163538	0.187295	0.294644	0.524994	rs113569514	1.151294	2.037033	0.57195
rs1169243	0.063649	0.26687	0.811491	rs116353863	4.129846	2.40756	0.086278
rs1170791	0.085948	0.235454	0.715087	rs11692435	-1.6483	2.155059	0.444359
rs1172767	-0.46033	0.32455	0.156087	rs117079142	-0.38789	2.144943	0.856493
rs1188459	-0.02378	0.280012	0.932333	rs11727676	-4.97971	3.192125	0.11876
rs1190375	-0.04196	0.142568	0.768535	rs11884596	0.924764	2.557625	0.717672
rs1214354	0.057431	0.241454	0.811992	rs11903757	-0.77698	1.350694	0.565123
rs1214431	0.375251	0.260233	0.149307	rs12143541	2.10051	2.267544	0.354271
rs1224100	-0.18304	0.219633	0.404618	rs12144319	-0.25357	2.471524	0.918284
rs1224663	-0.33951	0.319787	0.28838	rs12241008	-2.4089	2.074332	0.245524
rs1237271	-0.34972	0.185036	0.058757	rs12246635	-3.26912	2.669285	0.220682
rs1242760	0.069808	0.210228	0.739845	rs12372718	1.039956	1.745927	0.551411
rs1251451	-0.19568	0.189193	0.301009	rs12427600	-2.59592	1.993323	0.192812
rs1260352	-0.25023	0.588688	0.670796	rs12514517	1.41391	1.781342	0.427351
rs1263594	0.011543	0.212796	0.95674	rs12603526	2.868319	5.332484	0.590649
rs1267202	0.573585	0.3199	0.072971	rs12635946	-4.4186	2.019074	**0.028638**
rs1270849	0.190087	0.237383	0.423271	rs12672022	-1.23873	2.925864	0.672023
rs1281876	-0.23835	0.255356	0.350615	rs12708491	0.348884	2.188486	0.87334
rs1314935	0.173867	0.34011	0.609205	rs12818766	-1.4158	1.9489	0.467557
rs13831	-0.13341	0.229142	0.560436	rs13149359	0.441277	3.12358	0.887654
rs1391441	0.37733	0.380589	0.321472	rs13831	1.544678	2.24841	0.492077
rs1476570	-0.08531	0.148127	0.564684	rs1391441	0.355502	3.204949	0.911678
rs1535	-0.0209	0.247226	0.932618	rs1476570	-2.53634	1.581683	0.108809
rs1687881	-0.31338	0.287132	0.275092	rs1535	0.313737	2.609275	0.904294
rs1701114	0.407785	0.225766	0.070883	rs16878812	2.827405	3.238652	0.382653
rs1703528	-0.22117	0.22667	0.329191	rs17011141	4.485964	1.989958	**0.024177**
rs1709498	-0.20109	0.220367	0.361493	rs17035289	0.127164	1.90882	0.946885
rs1741640	-0.12331	0.172763	0.475398	rs17094983	-3.35401	1.883057	0.074888
rs174533	-0.03294	0.252059	0.896034	rs1741640	1.432823	1.524857	0.3474
rs174537	-0.0219	0.115065	0.849085	rs174533	-0.02554	2.599668	0.992161
rs1781646	-0.16604	0.195736	0.396277	rs174537	0.036122	1.193303	0.975851
rs1886450	0.096712	0.193055	0.616402	rs17816465	-1.15389	1.888846	0.541268
rs209489	-0.0181	0.049569	0.714942	rs1886450	0.647209	1.903161	0.733803
rs2179593	-0.58451	0.252828	0.020784	rs209489	0.969457	0.502242	0.053575
rs2423279	-0.20175	0.142238	0.156065	rs2179593	-0.33746	2.559318	0.895099
rs2427308	-0.11239	0.157705	0.476065	rs2423279	-0.5498	1.392352	0.692939
rs28488	-0.1568	0.328906	0.633563	rs2427308	2.818753	1.567528	0.072143
rs285245	-0.25259	0.27568	0.359545	rs28488	1.957133	2.953204	0.507513
rs2884075	-0.19819	0.16643	0.233709	rs285245	4.759294	2.906477	0.101531
rs3087967	-0.05741	0.150403	0.702683	rs28840750	1.43652	1.507447	0.340616
rs3217810	-0.08957	0.189322	0.636121	rs3087967	2.330125	1.460442	0.110602
rs3479759	-0.41753	0.300925	0.16529	rs3217810	0.945322	1.586197	0.551197
rs3547027	-0.09633	0.236984	0.684386	rs34797592	4.498148	2.857932	0.115507
rs3580816	-0.19719	0.312457	0.527977	rs35470271	0.79981	2.231346	0.720012
rs3731861	0.378366	0.276013	0.170429	rs35808169	-1.58483	2.963058	0.592745
rs3787089	-0.25374	0.247374	0.305009	rs3731861	0.008666	2.547328	0.997286
rs3801081	0.196996	0.214433	0.358262	rs3787089	-2.54956	2.337174	0.275328
rs3830041	0.134171	0.185186	0.468747	rs3801081	-1.56274	2.015046	0.438023
rs4313119	-0.02543	0.308552	0.934305	rs3830041	6.506349	1.866531	**0.000491**
rs4450168	-0.46824	0.272405	0.085631	rs4313119	-0.88459	2.827752	0.754414
rs448513	-0.27235	0.33353	0.414176	rs4450168	-1.16441	2.137757	0.585969
rs4759277	0.339249	0.323098	0.293724	rs448513	-6.12521	3.427125	0.073893
rs4776316	-0.05136	0.267707	0.847867	rs4759277	0.15398	3.075825	0.960074
rs4811050	0.258892	0.227331	0.254775	rs4776316	-2.06819	2.121723	0.329675

rs4813802	-0.15959	0.177463	0.368485	rs4811050	1.38663	2.03483	0.495588
rs4901473	-0.63121	0.33469	0.0593	rs4813802	-2.30217	1.661103	0.165769
rs4919687	-0.10618	0.132025	0.421243	rs4901473	1.392218	2.687711	0.604463
rs4944940	-0.13964	0.147396	0.343454	rs4919687	1.089383	1.194246	0.361667
rs5632496	-0.21857	0.245379	0.373071	rs4944940	-0.30884	1.480629	0.834773
rs6031311	-0.53104	0.305738	0.082404	rs56324967	0.523082	2.295641	0.819756
rs6061231	-0.06334	0.103459	0.540418	rs6031311	-1.72922	3.098908	0.576837
rs6063514	0.170207	0.232712	0.464531	rs6061231	2.128032	0.997738	**0.032936**
rs6085661	-0.19461	0.190664	0.3074	rs6085661	-2.22981	1.771108	0.208033
rs6240496	0.392559	0.321561	0.222164	rs62404966	5.68914	3.069733	0.063839
rs639933	-0.03495	0.27383	0.898433	rs639933	-2.78191	2.25071	0.216454
rs6933790	0.096402	0.223817	0.666673	rs6933790	1.435314	2.173745	0.509064
rs7013278	-0.28292	0.114073	**0.013133**	rs7013278	0.436757	1.073754	0.684187
rs7014346	-0.13006	0.090524	0.15078	rs7014346	0.40335	0.857816	0.638209
rs7160450	-0.65189	0.334158	0.051075	rs7160450	1.87407	2.627817	0.475743
rs7229639	0.113497	0.125204	0.364674	rs7229639	0.143806	1.294488	0.911544
rs7294248	-0.1085	0.257661	0.673684	rs72942485	1.354271	3.460296	0.695521
rs7306832	0.093397	0.360294	0.795463	rs73068325	0.66936	2.757667	0.808217
rs7320812	-0.05558	0.206886	0.788198	rs73208120	-2.59561	1.958092	0.184979
rs7333607	0.062985	0.232988	0.786902	rs7333607	-3.09052	2.233858	0.166514
rs7337693	0.082263	0.150563	0.584811	rs73376930	-2.33124	1.357784	0.085989
rs7397558	-0.31113	0.257685	0.227274	rs73975588	3.53362	2.310561	0.126182
rs7495132	0.055206	0.226466	0.807408	rs7495132	0.554312	2.264086	0.806589
rs7568686	-0.37843	0.253387	0.13531	rs75686861	-3.34099	2.363479	0.157482
rs7595492	-0.0483	0.231719	0.834879	rs75954926	3.545349	1.757531	**0.043671**
rs7777659	0.010212	0.258807	0.968524	rs77776598	-1.23027	2.346517	0.600073
rs7834100	-0.07306	0.287147	0.799151	rs78341008	-0.63643	2.602871	0.806835
rs7836858	0.024263	0.267713	0.927787	rs78368589	-1.75794	2.364299	0.457157
rs7993934	0.242135	0.206715	0.241458	rs7993934	0.27028	1.939033	0.889143
rs8000189	0.384596	0.273851	0.1602	rs8000189	0.007796	2.577876	0.997587
rs8020436	-0.19724	0.279017	0.47962	rs8020436	1.876987	2.481083	0.449338
rs899244	-0.02817	0.22449	0.900133	rs899244	0.743151	2.230391	0.738989
rs9271695	0.26855	0.22109	0.224494	rs9271695	-1.39143	2.048906	0.49707
rs9271770	0.334702	0.304375	0.27149	rs9271770	-1.06716	2.396797	0.656142
rs961253	-0.0072	0.140547	0.959167	rs961253	0.34955	1.322437	0.791531
rs983318	0.102441	0.32695	0.754036	rs983318	-3.47801	2.870485	0.225648
rs983402	0.277245	0.259789	0.285886	rs983402	0.434209	2.465465	0.860202
rs9924886	0.046079	0.302683	0.879	rs9924886	4.084854	2.784675	0.142402
All - Invers	-0.05283	0.018762	**0.004864**	All - Inverse va	0.203226	0.178734	0.255526
All - MR E	-0.06813	0.04151	0.103757	All - MR Egge	0.919172	0.405224	**0.025377**
**Kidney cancer & covid susceptibility**				**Kidney cancer & covid severity**			
rs1027643	-0.03973	0.079142	0.615695	rs1027643	0.079522	1.095354	0.942125
rs1181326	0.101078	0.174863	0.563238	rs11813268	-0.22909	1.797074	0.898563
rs2283873	0.011941	0.046863	0.798879	rs2283873	-0.15917	0.554149	0.773931
rs3755132	-0.08425	0.066236	0.203361	rs3755132	-0.24224	0.583265	0.677905
rs4903064	0.11483	0.097403	0.23843	rs4903064	-0.11199	0.903786	0.901383
rs5955543	0.014199	0.049617	0.774747	rs5955543	-0.27813	0.295886	0.347228
rs7491126	-0.15605	0.162295	0.336284	rs74911261	3.055971	1.796736	0.088972
All - Invers	-0.00341	0.026489	0.897616	All - Inverse va	-0.1775	0.221974	0.423923
All - MR E	-0.0224	0.045437	0.642913	All - MR Egge	-0.2742	0.359502	0.480045
**Gastric cancer & covid susceptibility**				**Gastric cancer & covid severity**			
rs1002900	0.089828	0.120905	0.457505	rs2294693	1.077912	1.080027	0.318259
rs2294693	-0.23963	0.108812	**0.027646**	rs7624041	4.081883	1.355941	**0.002609**
rs7624041	-0.00154	0.145614	0.991554				
All - Invers	-0.07082	0.104858	0.499404	All - Inverse va	2.243957	1.463935	0.125319
All - MR E	-0.60713	0.861834	0.609295	All - MR Egge	NA	NA	NA
**Pancreatic cancer & covid susceptibility**				**Pancreatic cancer & covid severity**			
rs1165523	-0.05884	0.098853	0.551708	rs11655237	-1.72903	0.996661	0.082771

rs1330301(	-0.20134	0.102236	**0.048907**	rs13303010	0.055056	1.095747	0.959927
rs1517037	0.01809	0.129659	0.88904	rs1517037	0.46421	1.145196	0.685217
rs1698682!	0.030876	0.180478	0.864162	rs16986825	-2.90072	1.404388	**0.038879**
rs1768860	-0.0053	0.142545	0.970341	rs17688601	0.58358	1.206571	0.628621
rs2736098	0.02779	0.103629	0.788568	rs2736098	0.990517	0.941421	0.29273
rs3522613	-0.01133	0.118411	0.92374	rs35226131	0.09433	1.01324	0.925826
rs4795218	-0.11456	0.152574	0.45273	rs4795218	-1.20133	1.332203	0.367184
rs5768709	-0.15623	0.078214	**0.045773**	rs5768709	0.663474	0.665043	0.318453
rs6971499	-0.04876	0.101286	0.63025	rs6971499	-0.10834	1.046502	0.917544
rs7190458	0.116975	0.09739	0.229714	rs7190458	-1.78708	1.154431	0.121618
rs7214041	-0.05294	0.100303	0.597614	rs7214041	-1.93821	0.991649	0.050638
rs9554197	0.0176	0.122683	0.885928	rs9554197	0.381467	1.193407	0.749237
rs9581943	0.066433	0.117865	0.573001	rs9581943	0.460905	1.105809	0.676822
rs962856	0.042054	0.141659	0.766568	rs962856	0.967363	1.45356	0.505722
rs9854771	0.298471	0.138312	**0.030931**	rs9854771	0.547541	1.317558	0.677723
All - Invers	-0.02097	0.02865	0.464218	All - Inverse va	-0.16968	0.287447	0.554997
All - MR E	-0.14979	0.090567	0.120392	All - MR Egge	-0.9958	0.939716	0.307232

Supplementary table 5. Heterogeneity test for each MR sub-analysis

MR_method	Glioma & covid susceptibility			Glioma & covid severity		
	**Q**	**Q_df**	**Q_pval**	**Q**	**Q_df**	**Q_pval**
MR Egger	15.58	12	0.21	20.39	12	0.06
IVW	15.99	13	0.25	20.43	13	0.085
	Squamous lung cancer & covid susceptibility			Squamous lung cancer & covid severity		
MR Egger	6.24	13	0.94	25.29	10	**0.005**
IVW	8.42	14	0.87	27.98	11	**0.003**
	Lung adenocarcinoma & covid susceptibility			Lung adenocarcinoma & covid severity		
MR Egger	13.97	14	0.45	17.29	14	0.24
IVW	14.21	15	0.51	17.3	15	0.3
	Melanoma & covid susceptibility			Melanoma & covid severity		
MR Egger	3.42	7	0.84	6.32	7	0.5
IVW	3.65	8	0.89	6.46	8	0.6
	Lymphoid leukemia & covid susceptibility			ymphoid leukemia & covid severi		
MR Egger	15.5	26	0.95	20.65	26	0.76
IVW	15.51	27	0.96	20.06	27	0.52
	Hepatocellular carcinoma & covid susceptibility			atocellular carcinoma & covid sev		
MR Egger	5.95	1	**0.015**	3.2	1	0.07
IVW	7.74	2	**0.021**	6.53	2	**0.04**
	Colorectal cancer & covid susceptibility			Colorectal cancer & covid severity		
MR Egger	90.84	104	0.82	104.72	104	0.46
IVW	91.01	105	0.83	108.6	105	0.39
	Kidney cancer & covid susceptibility			Kidney cancer & covid severity		
MR Egger	4.38	4	0.5	3.31	5	0.65
IVW	4.65	5	0.59	3.43	6	0.73
	Gastric cancer & covid susceptibility			Gastric cancer & covid severity		
MR Egger	3.15	1	0.08	\	\	\
IVW	4.4	2	0.11	3	1	0.08
	pancreatic cancer & covid susceptibility			pancreatic cancer & covid severity		
MR Egger	13.4	14	0.5	16.9	14	0.26
IVW	15.44	15	0.42	17.42	15	0.29

Supplementary table 6. Pleiotrophy test for each MR sub-analysis

Glioma & covid susceptibility			Glioma & covid severity		
egger_intercep	se	P_value	egger_intercept	se	P_value
0.006	0.012	0.585	0.018	0.119	0.884
Squamous lung cancer & covid			Squamous lung cancer & covid		
0.022	0.015	0.164	-0.309	0.3	0.327
Lung adenocarcinoma & covid			Lung adenocarcinoma & covid severity		
-0.018	0.036	0.635	-0.017	0.356	0.963
Melanoma & covid susceptibility			Melanoma & covid severity		
-0.01	0.02	0.648	-0.08	0.206	0.724
Lymphoid leukemia & covid susceptibility			Lymphoid leukemia & covid severity		
-0.0006	0.008	0.945	0.18	0.08	**0.03**
epatocellular carcinoma & covid susceptibil			epatocellular carcinoma & covid severi		
0.043	0.079	0.68	-0.59	0.57	0.49
Colorectal cancer & covid susceptibility			Colorectal cancer & covid severity		
0.002	0.004	0.68	-0.08	0.04	0.05
Kidney cancer & covid susceptibility			Kidney cancer & covid severity		
0.009	0.017	0.63	0.054	0.157	0.746
Gastric cancer & covid susceptibility			Gastric cancer & covid severity		
0.08	0.13	0.64	\	\	\
pancreatic cancer & covid susceptibility			ancreatic cancer & covid susceptibility		
0.023	0.016	0.175	0.11	0.167	0.52

Supplementary table 7. Sensitivity analysis for each MR sub-analysis by leave-one-out of every SNP.

Glioma & covid susceptibility				Glioma & covid severity			
**SNP**	**beta**	**se**	**P_value**	**SNP**	**beta**	**se**	**P_value**
rs10069690	0.00623185	0.01987984	0.75391913	rs10069690	0.099422	0.189515	0.599852805
rs10131032	0.00709958	0.01771021	0.6885123	rs10131032	-0.01267	0.187786	0.9462058
rs10842893	-0.007779	0.01752304	0.65709318	rs10842893	0.014348	0.204069	0.943945886
rs11233250	0.00478584	0.01811588	0.79164191	rs11233250	0.003047	0.184985	0.986856876
rs11599775	0.00377848	0.01796216	0.83338826	rs11599775	-0.01064	0.187201	0.954667613
rs12752552	0.0049698	0.01809641	0.78360106	rs12752552	-0.01411	0.187031	0.939876472
rs2297440	-0.004491	0.01700252	0.79167477	rs2297440	-0.06763	0.187858	0.718827571
rs3751667	0.00321054	0.01774327	0.85641166	rs3751667	0.008221	0.181618	0.963894859
rs3851634	0.00987788	0.01599816	0.53694543	rs3851634	-0.03035	0.186935	0.871006271
rs4252707	0.00195702	0.01701407	0.90842641	rs4252707	0.025218	0.170852	0.882655317
rs55705857	0.01897525	0.01892623	0.31605888	rs55705857	0.063572	0.207274	0.759069211
rs634537	0.00994455	0.01807827	0.58226231	rs634537	-0.0903	0.178309	0.612544077
rs7572263	0.00404682	0.01810978	0.82317698	rs7572263	-0.04843	0.174275	0.781071915
rs78378222	0.00675849	0.0181472	0.70957579	rs78378222	-0.07886	0.16408	0.630807424
All	0.00486926	0.01723825	0.77758444	All	-0.01187	0.178109	0.94685405
**Squamous lung cancer & covid susceptibility**				**Squamous lung cancer & covid severity**			
rs113855064	0.01177152	0.02532991	0.64212613	rs12686364	-0.21388	0.630735	0.734534067
rs11571818	0.01610161	0.02543003	0.5266207	rs13191296	0.088115	0.540938	0.870602186
rs12686364	0.01442888	0.02793698	0.60551938	rs13218875	0.124354	0.522828	0.811997665
rs13191296	0.00982835	0.0254524	0.69938828	rs13220495	0.0542	0.539137	0.919922549
rs13218875	0.00839693	0.02541498	0.741103	rs148696809	0.1397	0.523909	0.789738963
rs13220495	0.01000955	0.02527868	0.69212851	rs149949	0.063662	0.55001	0.907853567
rs148696809	0.0095412	0.02545643	0.7078055	rs34107459	0.063378	0.548852	0.908069968
rs149949	0.00892781	0.02553823	0.72664928	rs34878803	0.137992	0.520941	0.79109507
rs34107459	0.00663384	0.02544713	0.79433064	rs35768595	0.065045	0.537951	0.903760317
rs34878803	0.01166293	0.02549717	0.64736888	rs36109883	0.075988	0.54844	0.889803508
rs35768595	0.00982283	0.02540252	0.69898768	rs56404467	0.06155	0.561238	0.912672602
rs36109883	0.00906994	0.02554169	0.72251206	**rs8040868**	**-0.85825**	**0.420575**	**0.041284353**
rs56404467	0.01845951	0.0253442	0.46639789				
rs71603396	0.02464243	0.02898249	0.39518474				
rs8040868	0.02111854	0.02731979	0.43951513				
All	0.01241134	0.02501591	0.61979731	All	0.007796	0.519156	0.988019044
**Lung adenocarcinoma & covid susceptibility**				**Lung adenocarcinoma & covid severity**			
rs11591710	0.00955969	0.04160486	0.81826788	rs11591710	1.13519	0.435415	0.00913004
rs11632038	0.01350311	0.0410215	0.74202607	rs11632038	1.079194	0.442528	0.014740053
rs11639372	0.02164109	0.04382915	0.62147544	rs11639372	0.940549	0.460042	0.040905341
rs17038564	0.02035331	0.0402164	0.61279013	rs17038564	1.116794	0.413696	0.006943325
rs17400427	0.00746287	0.04116825	0.85615	rs17400427	1.094533	0.441677	0.013207308
rs2004038	0.03555215	0.04227817	0.40039823	rs2004038	0.929804	0.428422	0.029984187
rs2495239	0.01702931	0.04083227	0.67663803	rs2495239	1.055373	0.437241	0.015791021
rs28408315	0.01359318	0.04092347	0.7397677	rs28408315	1.077706	0.440074	0.014328495
rs28516445	0.00949781	0.04087711	0.81626596	rs28516445	1.099282	0.438631	0.012204755
rs34517439	0.00411303	0.04065121	0.91940869	rs34517439	0.96513	0.413902	0.019712119
rs4324798	-0.0016829	0.04067848	0.96699951	rs4324798	1.238672	0.388138	0.001416271
rs62560775	0.0158531	0.04134232	0.70137916	rs62560775	1.154629	0.433782	0.007773037
rs7086803	-0.0002889	0.04071472	0.99433942	rs7086803	1.007311	0.413156	0.014765242
rs7169304	0.01602002	0.0411997	0.69739575	rs7169304	1.001287	0.436596	0.021825179
rs7741164	-0.0003322	0.04032268	0.99342754	rs7741164	1.150386	0.384627	0.002781402
rs885518	0.00650023	0.0412896	0.87490572	rs885518	1.105926	0.438951	0.011753038
All	0.01157639	0.03978547	0.77107409	All	1.073719	0.41089	0.008971052
**Melanoma & covid susceptibility**				**Melanoma & covid severity**			
rs11591710	0.01973013	0.04726383	0.67635169	rs11591710	0.160907	0.439132	0.714051803
rs13016963	0.00900228	0.04968181	0.85621154	rs13016963	-0.05741	0.464613	0.901660357
rs1393350	0.03117661	0.04989593	0.53208077	rs1393350	0.285946	0.464982	0.538580246

rs16953002	0.0405387	0.0481755	0.40007931
rs187843643	0.02749073	0.04696483	0.55831437
rs4436359	0.02642202	0.04690644	0.57323607
rs6059655	-0.0007575	0.05250297	0.98848804
rs62389423	0.01186028	0.05036265	0.81382266
rs9745545	0.02179909	0.04788301	0.64892408
All	0.0213529	0.04596553	0.6422598

Lymphoid leukemia & covid susceptibility

rs10821936	0.00962568	0.00966327	0.31919688
rs11083846	0.00840509	0.00922558	0.36226204
rs113650570	0.00775439	0.00924456	0.4015782
rs11978267	0.00887337	0.00949618	0.35009002
rs11980379	0.00817957	0.01013633	0.41969201
rs12779301	0.00993328	0.00918903	0.27970027
rs13015798	0.00681364	0.00916548	0.45723778
rs13401811	0.00969978	0.00925099	0.2944027
rs17133805	0.00832299	0.00945291	0.37860583
rs17481869	0.00792552	0.00925339	0.39172199
rs17483466	0.00815312	0.00923432	0.37728201
rs210142	0.00712256	0.00927814	0.44268234
rs2296624	0.00914947	0.00919151	0.31952939
rs2953196	0.00936848	0.0092037	0.3087241
rs35837782	0.00983449	0.0091852	0.28430958
rs35923643	0.00865195	0.00936807	0.35571681
rs41271473	0.00930105	0.00916063	0.30994942
rs4245595	0.00970085	0.00944635	0.30444818
rs4982731	0.00885212	0.00925625	0.33890101
rs4987856	0.00748893	0.00918461	0.41485608
rs57214277	0.00860854	0.00915151	0.34687437
rs58055674	0.00835931	0.00923918	0.36558838
rs674313	0.01179046	0.0094822	0.21370878
rs71597109	0.00875895	0.00915389	0.33864056
rs7176508	0.00858543	0.00928438	0.35511254
rs72742684	0.00919782	0.00920059	0.31745618
rs735665	0.00851818	0.00925861	0.3575585
rs75777619	0.00824684	0.00916639	0.36828915
All	0.00875252	0.00913072	0.33777036

Hepatocellular cancer & covid susceptibility

rs17401966	0.15266377	0.04868664	**0.0017148**
rs7574865	0.03821316	0.07984401	0.63222488
rs9275319	0.00210934	0.03912039	0.95699957
All	0.0443657	0.0559558	0.42785336

Colorectal cancer & covid susceptibility

rs10049390	-0.051292	0.01879493	0.00635202
rs1035209	-0.0534527	0.01886641	0.00460819
rs10411210	-0.0516998	0.01889464	0.00621503
rs10506868	-0.0519746	0.01877521	0.00563571
rs10795668	-0.0521152	0.01897975	0.00603582
rs10821907	-0.0522751	0.01880331	0.00543419
rs10849432	-0.0549106	0.01887376	0.00362166
rs10849433	-0.0527622	0.01880128	0.00501128
rs10849438	-0.0532167	0.01882798	0.00470639
rs10980628	-0.0541145	0.01880022	0.00399705
rs11064437	-0.0545089	0.01878646	0.00371383
rs11087784	-0.0519878	0.01880037	0.00568781
rs11190164	-0.0523789	0.01883341	0.00541635
rs113569514	-0.0527104	0.01883025	0.00512229
rs116353863	-0.0538082	0.01879986	0.00420765

rs16953002	0.119853	0.455461	0.792437302
rs4436359	0.312405	0.440978	0.478674977
rs45430	0.195071	0.45212	0.66613582
rs6059655	-0.0007	0.468992	0.998814693
rs62389423	0.383774	0.456947	0.400983853
rs9745545	0.073631	0.455186	0.871494942
All	0.166123	0.42904	0.698610624

Lymphoid leukemia & covid severity

rs10821936	-0.08157	0.091082	0.370508635
rs11083846	-0.1152	0.087395	0.18747016
rs113650570	-0.07237	0.087281	0.407015169
rs11978267	-0.07704	0.089754	0.390682325
rs11980379	-0.07617	0.09597	0.427360579
rs12779301	-0.0986	0.086951	0.256781466
rs13015798	-0.08704	0.086935	0.316712712
rs13401811	-0.09972	0.087586	0.254913237
rs17133805	-0.08236	0.089515	0.357509962
rs17481869	-0.09339	0.087939	0.288225677
rs17483466	-0.08778	0.087624	0.316449989
rs210142	-0.07402	0.087544	0.397805701
rs2296624	-0.09596	0.086997	0.270021967
rs2953196	-0.09054	0.087114	0.298658006
rs35837782	-0.09855	0.086923	0.256883314
rs35923643	-0.08257	0.088847	0.35270517
rs41271473	-0.08943	0.086687	0.302241725
rs4245595	-0.08119	0.089546	0.364562962
rs4982731	-0.08582	0.087851	0.328632996
rs4987856	-0.08543	0.087278	0.327649705
rs57214277	-0.08337	0.086618	0.335807233
rs58055674	-0.09416	0.087655	0.282705284
rs674313	-0.01907	0.08917	0.830698346
rs71597109	-0.09079	0.086686	0.29494853
rs7176508	-0.10624	0.087828	0.226401197
rs72742684	-0.09702	0.087013	0.264860465
rs735665	-0.07351	0.087489	0.400758379
rs75777619	-0.08884	0.086767	0.30588468
All	-0.0862	0.08641	0.318465179

Hepatocellular cancer & covid severity

rs17401966	0.095511	1.187487	0.935894036
rs7574865	0.157068	0.353031	0.65638253
rs9275319	-0.25937	0.609508	0.670443149
All	-0.00862	0.481684	0.985718991

Colorectal cancer & covid severity

rs10049390	0.199652	0.179869	0.267005378
rs1035209	0.20549	0.180529	0.255010757
rs10411210	0.206653	0.180663	0.252681579
rs10506868	0.20148	0.179665	0.262110538
rs10774214	0.210584	0.181931	0.247071942
rs10795668	0.203552	0.182051	0.263522219
rs10821907	0.218209	0.179055	0.222969747
rs10849432	0.198091	0.180399	0.272175096
rs10849433	0.213819	0.179225	0.232860981
rs10849438	0.223576	0.179086	0.211875276
rs10980628	0.208582	0.17981	0.246042577
rs11064437	0.202672	0.179604	0.259136015
rs11087784	0.214842	0.179419	0.231137911
rs11190164	0.208739	0.180186	0.246673996
rs113569514	0.196116	0.180082	0.276137459

rs11692435	-0.0534092	0.01880825	0.00451605	rs116353863	0.182191	0.177841	0.30561804
rs117079142	-0.0537174	0.01882156	0.00431676	rs11692435	0.215622	0.179574	0.229852465
rs11727676	-0.0514643	0.01879314	0.00617274	rs117079142	0.207221	0.180134	0.249990383
rs11884596	-0.0529616	0.01880397	0.00485478	rs11727676	0.218984	0.177661	0.217727887
rs11903757	-0.0530223	0.01892631	0.0050864	rs11884596	0.199803	0.179951	0.2668605
rs12143541	-0.0535004	0.01881861	0.00446978	rs11903757	0.220106	0.180684	0.223154097
rs12144319	-0.0550673	0.01881066	0.0034175	rs12143541	0.191761	0.179548	0.285513138
rs12241008	-0.0518735	0.01883054	0.00587366	rs12144319	0.205547	0.180019	0.253532532
rs12246635	-0.0518404	0.01879408	0.00580962	rs12241008	0.222111	0.178909	0.214430359
rs12372718	-0.0497466	0.0188589	0.00834377	rs12246635	0.218344	0.178568	0.221425707
rs12427600	-0.0538152	0.01883687	0.00427789	rs12372718	0.194661	0.180315	0.280337989
rs12514517	-0.0514119	0.01885465	0.00639616	rs12427600	0.225155	0.178636	0.207521678
rs12603526	-0.0526299	0.01877124	0.00505117	rs12514517	0.191326	0.180084	0.288041535
rs12635946	-0.0533349	0.01883506	0.00463035	rs12603526	0.200328	0.179482	0.264359861
rs12672022	-0.0549927	0.01879406	0.00343275	rs12635946	0.238509	0.176413	0.17637684
rs12708491	-0.0543576	0.01882058	0.00387456	rs12672022	0.208447	0.179714	0.24609711
rs12818766	-0.0518237	0.01881256	0.00587387	rs12708491	0.202281	0.18017	0.261555083
rs13149359	-0.0535226	0.01879032	0.0043938	rs12818766	0.216499	0.179748	0.228410642
rs13831	-0.0522868	0.01882492	0.00547735	rs13149359	0.20247	0.179872	0.260319264
rs1391441	-0.0538785	0.01878455	0.00412767	rs13831	0.19498	0.179845	0.278296515
rs1476570	-0.0523011	0.01891404	0.00568876	rs1391441	0.202767	0.17986	0.259591303
rs1535	-0.0530156	0.01881597	0.00483866	rs1476570	0.237471	0.178165	0.182575131
rs16878812	-0.0517134	0.01880189	0.00595157	rs1535	0.202722	0.179999	0.260062392
rs17011141	-0.0560338	0.01882683	0.00291777	rs16878812	0.195476	0.17931	0.275645183
rs17035289	-0.0516693	0.01882631	0.00605978	rs17011141	0.16956	0.176433	0.336529079
rs17094983	-0.0517481	0.01883008	0.00599304	rs17035289	0.203876	0.180356	0.258303804
rs1741640	-0.0519896	0.01887333	0.00587537	rs17094983	0.234483	0.177364	0.186154138
rs174533	-0.0529414	0.0188139	0.00489362	rs1741640	0.186673	0.180247	0.30036397
rs174537	-0.0536755	0.0190162	0.00476327	rs174533	0.204276	0.179997	0.256422009
rs17816465	-0.0517808	0.0188485	0.0060104	rs174537	0.206931	0.181555	0.254381405
rs1886450	-0.0542565	0.01885094	0.00399973	rs17816465	0.215077	0.179941	0.231983546
rs209489	-0.0586374	0.02026971	0.00381755	rs1886450	0.199408	0.180317	0.268780624
rs2179593	-0.0498866	0.01881358	0.0080105	rs209489	0.096317	0.189356	0.610994633
rs2423279	-0.0501937	0.01892708	0.00800285	rs2179593	0.205788	0.179979	0.252874008
rs2427308	-0.0519756	0.0188959	0.00594812	rs2423279	0.215417	0.180792	0.233448456
rs28488	-0.0524912	0.01879231	0.00521847	rs2427308	0.169931	0.17837	0.340746741
rs285245	-0.0519011	0.01880531	0.00578158	rs28488	0.196993	0.179617	0.272755779
rs28840750	-0.0509595	0.01888207	0.00695833	rs285245	0.186507	0.177866	0.294370357
rs3087967	-0.0527582	0.01890941	0.00526995	rs28840750	0.186233	0.180259	0.30153847
rs3217810	-0.0524662	0.01885452	0.00539111	rs3087967	0.171975	0.179105	0.336959613
rs34797592	-0.0514074	0.01879828	0.00624393	rs3217810	0.194003	0.18052	0.282512167
rs35470271	-0.0525563	0.01882078	0.00523092	rs34797592	0.186924	0.178044	0.293777073
rs35808169	-0.0523082	0.01879562	0.00538586	rs35470271	0.199502	0.180091	0.267955772
rs3731861	-0.0548322	0.0188052	0.00354779	rs35808169	0.209538	0.179605	0.24334751
rs3787089	-0.0516682	0.0188159	0.00603291	rs3731861	0.204157	0.180016	0.25675052
rs3801081	-0.0547579	0.01883394	0.00364442	rs3787089	0.21888	0.178941	0.221255944
rs3830041	-0.05477	0.01885874	0.00368169	rs3801081	0.216762	0.179635	0.227555299
rs4313119	-0.0529323	0.01879649	0.00486146	rs3830041	0.146848	0.176528	0.405482608
rs4450168	-0.0508507	0.01880637	0.00685295	rs4313119	0.207444	0.179816	0.24864571
rs448513	-0.0521338	0.01879146	0.00553149	rs4450168	0.212532	0.179859	0.237341921
rs4759277	-0.0541571	0.01879342	0.00395526	rs448513	0.219912	0.176975	0.214010298
rs4776316	-0.0528379	0.01880795	0.00496436	rs4759277	0.203387	0.179885	0.258201928
rs4811050	-0.0549684	0.01882593	0.0035023	rs4776316	0.218918	0.179251	0.221975198
rs4813802	-0.0516238	0.01886745	0.00621662	rs4811050	0.194332	0.179982	0.280261793
rs4901473	-0.0510074	0.01879126	0.00663917	rs4813802	0.231588	0.178682	0.194945066
rs4919687	-0.051731	0.01895407	0.00634726	rs4901473	0.198121	0.179814	0.270544121
rs4944940	-0.051401	0.01891557	0.00657989	rs4919687	0.183611	0.181097	0.310639488
rs56324967	-0.051856	0.01881679	0.00585423	rs4944940	0.210543	0.180769	0.244136815

rs6031311	-0.051023	0.01879713	0.00663951
rs6061231	-0.0524734	0.01907803	0.00595112
rs6063514	-0.0542898	0.01882298	0.00392371
rs6085661	-0.0514444	0.01885321	0.00635885
rs62404966	-0.054352	0.01879373	0.00382762
rs639933	-0.0529149	0.0188059	0.00489686
rs6933790	-0.0538867	0.01882798	0.00420906
rs7013278	-0.0464335	0.01902074	0.01463834
rs7014346	-0.0493642	0.01917813	0.01005354
rs7160450	-0.0509362	0.01879135	0.00671581
rs7229639	-0.0566512	0.01897597	0.00283189
rs72942485	-0.0525339	0.01881165	0.00522822
rs73068325	-0.0532282	0.0187872	0.00460821
rs73208120	-0.0528078	0.01883934	0.005062
rs7333607	-0.0535865	0.01882284	0.00441487
rs73376930	-0.0549614	0.01890909	0.00365365
rs73975588	-0.051454	0.01881164	0.00623378
rs7495132	-0.0535772	0.01882643	0.00442927
rs75686861	-0.0510357	0.01881335	0.00667299
rs75954926	-0.0528605	0.01882351	0.00498166
rs77776598	-0.0531637	0.0188112	0.00471081
rs78341008	-0.0527439	0.01880189	0.00502786
rs78368589	-0.0532111	0.01880795	0.00466671
rs7993934	-0.0552806	0.01883947	0.00334303
rs8000189	-0.0548935	0.0188059	0.00351213
rs8020436	-0.0521747	0.01880427	0.00552663
rs899244	-0.0530041	0.01882758	0.00487415
rs9271695	-0.0551617	0.01882963	0.00339488
rs9271770	-0.0543087	0.01879745	0.00386285
rs961253	-0.0536586	0.01893114	0.00459107
rs983318	-0.0533436	0.01879268	0.00453216
rs983402	-0.0545612	0.01881083	0.00372545
rs9924886	-0.0532121	0.01879786	0.00464384
All	-0.0528306	0.01876171	0.00486447

Kidney cancer & covid susceptibility

rs1027643	0.00117328	0.02810991	0.96670678
rs11813268	-0.0058623	0.02679792	0.82683606
rs2283873	-0.0106147	0.0321103	0.74097002
rs3755132	0.01198281	0.02890029	0.67841591
rs4903064	-0.0128513	0.02752609	0.64058862
rs5955543	-0.0104271	0.0313264	0.73924532
rs74911261	0.00076907	0.02684869	0.97714795
All	-0.0034084	0.02648867	0.89761563

Gastric cancer & covid susceptibility

rs10029005	-0.1543215	0.11416721	0.17646647
rs2294693	0.05254186	0.09301999	0.57217928
rs7624041	-0.0921992	0.16382081	0.57356737
All	-0.0708243	0.10485845	0.49940411

Pancreatic cancer & covid susceptibility

rs11655237	-0.0175959	0.03078704	0.56763601
rs13303010	-0.0060337	0.02941899	0.83749707
rs1517037	-0.022919	0.03029233	0.44929246
rs16986825	-0.0222735	0.02994343	0.45696525
rs17688601	-0.0216109	0.03024401	0.4748862
rs2736098	-0.0248903	0.03058435	0.41574597
rs35226131	-0.0215517	0.03053155	0.48026075
rs4795218	-0.0176414	0.02979375	0.55377211
rs5768709	-0.0006401	0.03032198	0.98315728

rs56324967	0.20134	0.180104	0.263604264
rs6031311	0.209461	0.179558	0.243396008
rs6061231	0.141595	0.179189	0.429411315
rs6085661	0.22742	0.178892	0.2036307
rs62404966	0.185186	0.177213	0.296026437
rs639933	0.221538	0.178668	0.214995414
rs6933790	0.19512	0.179913	0.278133064
rs7013278	0.196798	0.182006	0.279575453
rs7014346	0.194458	0.183435	0.289103155
rs7160450	0.195719	0.179658	0.275976782
rs7229639	0.204342	0.181268	0.259619401
rs72942485	0.200249	0.179732	0.265211938
rs73068325	0.201325	0.179934	0.263188585
rs73208120	0.225955	0.178601	0.205821877
rs7333607	0.223739	0.178326	0.209601279
rs73376930	0.24641	0.178136	0.166582043
rs73975588	0.183847	0.178372	0.302685018
rs7495132	0.201098	0.180115	0.264209215
rs75686861	0.222931	0.178205	0.21094182
rs75954926	0.169471	0.177435	0.339518818
rs77776598	0.211312	0.179786	0.239852378
rs78341008	0.207071	0.179916	0.249758345
rs78368589	0.214122	0.179515	0.232955178
rs7993934	0.202671	0.180333	0.261067795
rs8000189	0.204139	0.180006	0.256765764
rs8020436	0.194786	0.179664	0.278291128
rs899244	0.199853	0.180103	0.26714516
rs9271695	0.215045	0.179749	0.231553688
rs9271770	0.210093	0.179842	0.24272195
rs961253	0.200595	0.181188	0.268246189
rs983318	0.217077	0.178556	0.224086586
rs983402	0.202046	0.180042	0.261770168
rs9924886	0.187704	0.178327	0.292531934
All	0.203226	0.178734	0.255526073

Kidney cancer & covid severity

rs1027643	-0.1885	0.226677	0.405634733
rs11813268	-0.1767	0.223687	0.429565004
rs2283873	-0.181	0.242259	0.45498219
rs3755132	-0.16653	0.240037	0.487821279
rs4903064	-0.1817	0.228988	0.427484284
rs5955543	-0.04796	0.335709	0.886403037
rs74911261	-0.22761	0.223688	0.308889138
All	-0.1775	0.221974	0.423923491

Gastric cancer & covid severity

All	2.243957	1.463935	0.125318738

Pancreatic cancer & covid severity

rs11655237	-0.0486	0.284374	0.864295515
rs13303010	-0.18392	0.306424	0.548360105
rs1517037	-0.20627	0.303125	0.496211567
rs16986825	-0.06684	0.272515	0.80623688
rs17688601	-0.20862	0.301497	0.488966833
rs2736098	-0.2716	0.295154	0.357477042
rs35226131	-0.18946	0.307831	0.538246699
rs4795218	-0.12633	0.298192	0.67182909
rs5768709	-0.33052	0.306917	0.281518395

rs6971499	-0.0186214	0.03080085	0.54546271	rs6971499	-0.17397	0.307729	0.571856679
rs7190458	-0.0336637	0.02954401	0.2545191	rs7190458	-0.07789	0.286959	0.786059668
rs7214041	-0.0182098	0.03079755	0.55433672	rs7214041	-0.03086	0.277826	0.911556792
rs9554197	-0.0231324	0.03037211	0.44627973	rs9554197	-0.19884	0.303319	0.512120429
rs9581943	-0.026305	0.02996298	0.3799882	rs9581943	-0.20888	0.303571	0.491409146
rs962856	-0.0235837	0.03006096	0.43273018	rs962856	-0.20954	0.297119	0.480656407
rs9854771	-0.0348981	0.02888157	0.22692568	rs9854771	-0.20052	0.301141	0.505502429
All	-0.0209693	0.02864974	0.46421822	All	-0.16968	0.287447	0.554996893

Supplementary table 8. A summary of genes associated with COVID-19.

Gene_symb	Ensembl ID	Full_name	Role in COVID-19
APOE	ENSG00000130203	apolipoprotein E	COVID-19 susceptibility
SLC6A20	ENSG00000163817	solute carrier family 6 member	COVID-19 susceptibility and severity
LZTFL1	ENSG00000163818	leucine zipper transcription fac	COVID-19 severity
CCR9	ENSG00000173585	C-C motif chemokine receptor	COVID-19 severity
FYCO1	ENSG00000163820	FYVE and coiled-coil domain a	COVID-19 severity
CXCR6	ENSG00000172215	C-X-C motif chemokine recept	COVID-19 severity
XCR1	ENSG00000173578	X-C motif chemokine receptor	COVID-19 severity
ABO	ENSG00000175164	alpha 1-3-N-acetylgalactosamir	COVID-19 severity
ERAP2	ENSG00000164308	endoplasmic reticulum aminope	COVID-19 death risk
BRF2	ENSG00000104221	RNA polymerase III transcripti	COVID-19 death risk
TMEM181	ENSG00000146433	transmembrane protein 181	COVID-19 death risk
ALOXE3	ENSG00000179148	arachidonate lipoxygenase 3	COVID-19 death risk
ACE2	ENSG00000130234	angiotensin I converting enzym	COVID-19 Receptor
ANPEP	ENSG00000166825	alanyl aminopeptidase, membra	COVID-19 Receptor
DPP4	ENSG00000197635	dipeptidyl peptidase 4	COVID-19 Receptor
ENPEP	ENSG00000138792	glutamyl aminopeptidase	COVID-19 Receptor
TMPRSS2	ENSG00000184012	transmembrane serine protease	COVID-19 Receptor

Supplementary table 9.The mutual exclusion and co-occurrence of gene-pairs associated with COVID-19.

A	B	Neithe	A No	B No	Both	Log2 (	p-Value	q-Value	Tendency
LZTFL1	CCR9	4634	21	34	36	>3	<0.001	<0.001	Co-occurrence
LZTFL1	CXCR6	4651	27	17	30	>3	<0.001	<0.001	Co-occurrence
CCR9	CXCR6	4639	39	16	31	>3	<0.001	<0.001	Co-occurrence
CXCR6	XCR1	4634	18	44	29	>3	<0.001	<0.001	Co-occurrence
CCR9	XCR1	4614	38	41	32	>3	<0.001	<0.001	Co-occurrence
CCR9	FYCO1	4570	33	85	37	>3	<0.001	<0.001	Co-occurrence
LZTFL1	XCR1	4625	27	43	30	>3	<0.001	<0.001	Co-occurrence
SLC6A20	CXCR6	4630	48	20	27	>3	<0.001	<0.001	Co-occurrence
SLC6A20	CCR9	4610	45	40	30	>3	<0.001	<0.001	Co-occurrence
LZTFL1	FYCO1	4578	25	90	32	>3	<0.001	<0.001	Co-occurrence
FYCO1	XCR1	4564	88	39	34	>3	<0.001	<0.001	Co-occurrence
FYCO1	CXCR6	4585	93	18	29	>3	<0.001	<0.001	Co-occurrence
SLC6A20	FYCO1	4562	41	88	34	>3	<0.001	<0.001	Co-occurrence
SLC6A20	LZTFL1	4620	48	30	27	>3	<0.001	<0.001	Co-occurrence
SLC6A20	XCR1	4605	47	45	28	>3	<0.001	<0.001	Co-occurrence
ANPEP	ENPEP	4446	130	122	27	2.92	<0.001	<0.001	Co-occurrence
CCR9	ANPEP	4513	55	142	15	>3	<0.001	<0.001	Co-occurrence
FYCO1	ENPEP	4472	104	131	18	2.563	<0.001	<0.001	Co-occurrence
FYCO1	DPP4	4513	107	90	15	2.813	<0.001	<0.001	Co-occurrence
ERAP2	ANPEP	4490	78	141	16	2.708	<0.001	<0.001	Co-occurrence
ALOXE3	TMPRSS2	4570	89	55	11	>3	<0.001	<0.001	Co-occurrence
CCR9	ENPEP	4519	57	136	13	2.922	<0.001	<0.001	Co-occurrence
ANPEP	DPP4	4479	141	89	16	2.514	<0.001	<0.001	Co-occurrence
LZTFL1	TMEM181	4612	49	56	8	>3	<0.001	<0.001	Co-occurrence
ACE2	ENPEP	4477	99	134	15	2.34	<0.001	<0.001	Co-occurrence
SLC6A20	ANPEP	4505	63	145	12	2.565	<0.001	<0.001	Co-occurrence
CCR9	ACE2	4551	60	104	10	2.867	<0.001	<0.001	Co-occurrence
FYCO1	ANPEP	4461	107	142	15	2.139	<0.001	<0.001	Co-occurrence
ALOXE3	ENPEP	4489	87	136	13	2.302	<0.001	<0.001	Co-occurrence
LZTFL1	ANPEP	4521	47	147	10	2.71	<0.001	<0.001	Co-occurrence
DPP4	ENPEP	4484	92	136	13	2.22	<0.001	<0.001	Co-occurrence
TMEM181	ENPEP	4522	54	139	10	2.591	<0.001	<0.001	Co-occurrence
FYCO1	TMEM181	4548	113	55	9	2.719	<0.001	<0.001	Co-occurrence
ERAP2	TMEM181	4575	86	56	8	2.926	<0.001	<0.001	Co-occurrence
TMEM181	ALOXE3	4569	56	92	8	2.827	<0.001	<0.001	Co-occurrence
LZTFL1	ACE2	4562	49	106	8	2.813	<0.001	<0.001	Co-occurrence
LZTFL1	ENPEP	4528	48	140	9	2.6	<0.001	<0.001	Co-occurrence
SLC6A20	ACE2	4545	66	105	9	2.561	<0.001	<0.001	Co-occurrence
TMEM181	DPP4	4564	56	97	8	2.749	<0.001	<0.001	Co-occurrence
ALOXE3	ANPEP	4480	88	145	12	2.075	<0.001	<0.001	Co-occurrence
SLC6A20	ENPEP	4511	65	139	10	2.32	<0.001	<0.001	Co-occurrence
CXCR6	ACE2	4571	40	107	7	2.902	<0.001	<0.001	Co-occurrence
FYCO1	ACE2	4500	111	103	11	2.114	<0.001	<0.001	Co-occurrence
ERAP2	ENPEP	4493	83	138	11	2.109	<0.001	<0.001	Co-occurrence
TMEM181	ANPEP	4513	55	148	9	2.319	<0.001	<0.001	Co-occurrence
CCR9	TMPRSS2	4595	64	60	6	2.844	<0.001	0.001	Co-occurrence
ALOXE3	ACE2	4520	91	105	9	2.09	<0.001	0.002	Co-occurrence
CCR9	ALOXE3	4562	63	93	7	2.446	<0.001	0.002	Co-occurrence
BRF2	ENPEP	4403	173	134	15	1.51	<0.001	0.002	Co-occurrence
TMEM181	ACE2	4554	57	107	7	2.386	<0.001	0.002	Co-occurrence
APOE	ANPEP	4515	53	149	8	2.193	<0.001	0.002	Co-occurrence
BRF2	DPP4	4444	176	93	12	1.704	<0.001	0.002	Co-occurrence
BRF2	TMEM181	4482	179	55	9	2.035	<0.001	0.002	Co-occurrence
ACE2	DPP4	4515	105	96	9	2.011	<0.001	0.002	Co-occurrence
ERAP2	DPP4	4534	86	97	8	2.12	0.001	0.003	Co-occurrence

ACE2	ANPEP	4465	103	146	11	1.708	0.001	0.003	Co-occurrence
BRF2	ANPEP	4395	173	142	15	1.424	0.001	0.003	Co-occurrence
FYCO1	TMPRSS2	4544	115	59	7	2.229	0.001	0.003	Co-occurrence
ERAP2	ACE2	4525	86	106	8	1.99	0.002	0.004	Co-occurrence
CCR9	TMEM181	4596	65	59	5	2.583	0.002	0.005	Co-occurrence
CCR9	ERAP2	4567	64	88	6	2.283	0.002	0.006	Co-occurrence
FYCO1	ERAP2	4517	114	86	8	1.882	0.003	0.006	Co-occurrence
CXCR6	ALOXE3	4583	42	95	5	2.522	0.003	0.006	Co-occurrence
SLC6A20	TMEM181	4591	70	59	5	2.475	0.003	0.007	Co-occurrence
ERAP2	ALOXE3	4538	87	93	7	1.973	0.004	0.007	Co-occurrence
CXCR6	TMPRSS2	4616	43	62	4	2.792	0.004	0.008	Co-occurrence
ENPEP	TMPRSS2	4517	142	59	7	1.916	0.004	0.009	Co-occurrence
XCR1	DPP4	4553	67	99	6	2.042	0.005	0.011	Co-occurrence
SLC6A20	DPP4	4551	69	99	6	1.999	0.006	0.012	Co-occurrence
CCR9	BRF2	4475	62	180	8	1.682	0.006	0.012	Co-occurrence
XCR1	ACE2	4544	67	108	6	1.914	0.008	0.015	Co-occurrence
LZTFL1	DPP4	4568	52	100	5	2.135	0.008	0.016	Co-occurrence
APOE	TMEM181	4604	57	60	4	2.429	0.009	0.017	Co-occurrence
XCR1	ANPEP	4502	66	150	7	1.67	0.01	0.019	Co-occurrence
APOE	CCR9	4598	57	66	4	2.29	0.012	0.022	Co-occurrence
BRF2	ACE2	4433	178	104	10	1.26	0.015	0.026	Co-occurrence
APOE	ACE2	4555	56	109	5	1.9	0.015	0.027	Co-occurrence
XCR1	TMEM181	4592	69	60	4	2.149	0.017	0.029	Co-occurrence
BRF2	ALOXE3	4446	179	91	9	1.297	0.017	0.03	Co-occurrence
CXCR6	ANPEP	4526	42	152	5	1.826	0.019	0.032	Co-occurrence
APOE	FYCO1	4547	56	117	5	1.795	0.02	0.033	Co-occurrence
SLC6A20	TMPRSS2	4588	71	62	4	2.06	0.02	0.033	Co-occurrence
SLC6A20	ALOXE3	4555	70	95	5	1.776	0.021	0.034	Co-occurrence
ANPEP	TMPRSS2	4508	151	60	6	1.578	0.021	0.035	Co-occurrence
LZTFL1	BRF2	4486	51	182	6	1.536	0.025	0.039	Co-occurrence
CXCR6	TMEM181	4617	44	61	3	2.368	0.025	0.04	Co-occurrence
LZTFL1	ALOXE3	4572	53	96	4	1.846	0.032	0.05	Co-occurrence

Supplementary table 10. Summary of SNPs used for MR analysis in terms of lung adenocarcinoma and COVID-19 severity.

SNP	effect allele	eaf	beta	se	F
rs2495239	A	0.382	0.173953	0.030047	1129.692
rs17038564	G	0.161	0.139762	0.024314	266.4905
rs7741164	A	0.34	0.173953	0.023499	175.7547
rs7086803	A	0.28	0.165514	0.023697	1787.028
rs4324798	A	0.09	0.14842	0.032891	109.4335
rs7741164	A	0.308	0.165514	0.023697	53.83905
rs7169304	G	0.72238	0.126314	0.017909	527.1298
rs11632038	A	0.794887	0.125224	0.020239	1267.155
rs17400427	T	0.743917	0.116585	0.020419	856.2971
rs28408315	G	0.72269	0.109177	0.018229	2629.362
rs2004038	A	0.418658	0.137518	0.017503	902.1949
rs11591710	C	0.136759	0.150558	0.023033	1169.368
rs34517439	A	0.104562	0.152912	0.027836	540.0263
rs885518	G	0.101095	0.154547	0.025293	208.9472
rs62560775	G	0.106544	0.165878	0.027739	386.7976
rs11639372	T	0.403533	0.172567	0.016389	4022.193
rs28516445	A	0.735247	0.108414	0.020602	1728.809
