# Supplementary material for: Cancer and COVID-19 Susceptibility and Severity: A Two-Sample Mendelian Randomization and Bioinformatic Analysis
Source: Front Cell Dev Biol. 2022 Jan 24;9:759257. doi: 10.3389/fcell.2021.759257 (PMC8818950; doi:10.3389/fcell.2021.759257)

**Supplementary figure1.** The genetic predisposition to lung adenocarcinoma is causally related to increased COVID-19 severity based on hospitalized and non-hospitalized cohort.


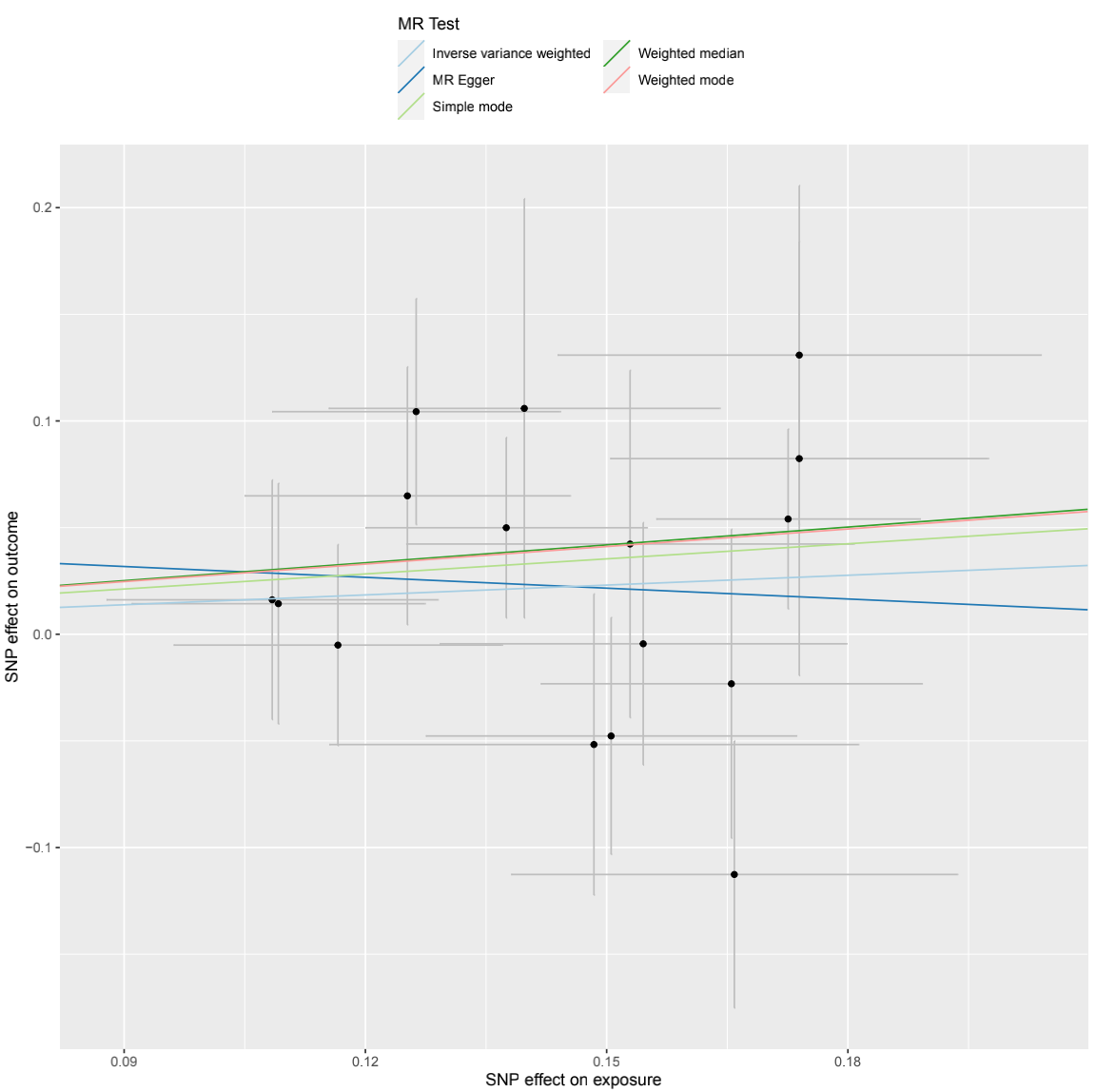

Supplement: Supplementary file 3 [file DataSheet2.docx]
